# The Nanosized Dye Adsorbents for Water Treatment

**DOI:** 10.3390/nano10020295

**Published:** 2020-02-10

**Authors:** Shahin Homaeigohar

**Affiliations:** Nanochemistry and Nanoengineering, Department of Chemistry and Materials Science, School of Chemical Engineering, Aalto University, Kemistintie 1, 00076 Aalto, Finland; shahin.homaeigohar@fau.de; Tel.: +49-152-025-79933

**Keywords:** dye pollution, nanomaterials, adsorption, water treatment, decolorization

## Abstract

Clean water is a vital element for survival of any living creature and, thus, crucially important to achieve largely and economically for any nation worldwide. However, the astonishingly fast trend of industrialization and population growth and the arisen extensive water pollutions have challenged access to clean water across the world. In this regard, 1.6 million tons of dyes are annually consumed. Thereof, 10%–15% are wasted during use. To decolorize water streams, there is an urgent need for the advanced remediation approaches involving utilization of novel materials and technologies, which are cost and energy efficient. Nanomaterials, with their outstanding physicochemical properties, can potentially resolve the challenge of need to water treatment in a less energy demanding manner. In this review, a variety of the most recent (from 2015 onwards) opportunities arisen from nanomaterials in different dimensionalities, performances, and compositions for water decolorization is introduced and discussed. The state-of-the-art research studies are presented in a classified manner, particularly based on structural dimensionality, to better illustrate the current status of adsorption-based water decolorization using nanomaterials. Considering the introduction of many newly developed nano-adsorbents and their classification based on the dimensionality factor, which has never been employed for this sake in the related literature, a comprehensive review will be presented.

## 1. Introduction

**Figure 1:**
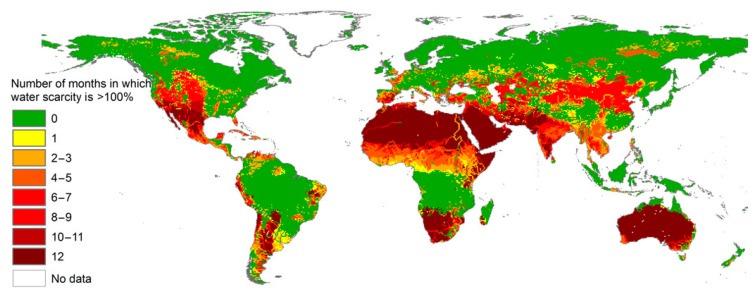
Water scarcity recorded for the world between 1996 and 2005. Reproduced with permission [1]. Copyright 2016, The American Association for the Advancement of Science (AAAS).

For many centuries, the human being had access to water as a plentiful, free resource across the world. This situation has drastically changed over recent decades and, currently, water scarcity has become a threat for sustainable development of the human community [1,2]. This challenge is going to exponentially expand and is, thus, considered a global systemic risk. Mekonen and Hoekstra [1] determined month-by-month blue water shortage statistics worldwide at a high spatial resolution. According to this assessment, they claimed that 4 billion people, i.e., two-thirds of the world’s population, with half living in India and China, suffer from intense water scarcity at least one month per year. Additionally, they stated that 0.5 billion people in the world are struggling with such a problem all year round (Figure 1).

The main causes of water scarcity are the urban, agricultural, and industrial pollutions. In these sectors, water consumption has raised as much as 70 (agriculture), 22 (industry), and 8% (domestic) of the available fresh water and, proportionally, a huge amount of wastewater containing a variety of pollutants has been generated [3]. Undoubtedly, the release of wastewater from commercial and industrial sectors alongside the untreated domestic sewage, and chemical pollutants into the fresh water resources is disastrous not only to the human community but also to animals and plants as well as to the ecosystem. Heavy metal ions, organics (e.g., dyes), and oils whose presence in any water stream disqualifies it for drinking are part of the major water contaminants [4].

Dyes have a history of thousands of years application for textile, paint, pigment, etc. Currently, almost 100,000 types of dyes are produced commercially. In terms of consumption volume, 1.6 million tons of dyes are annually consumed. Thereof, 10%–15% are wasted during use [5]. A variety of industries including those producing textile, leather, paper, cosmetics, plastics, food, etc. deal with a dyeing process involving organic dyes and water. Such processes create dye containing wastewaters, i.e., water streams contaminated with dye molecules that are colored organics with poor biodegradability [6,7,8,9]. Dyes, particularly azo dyes, are recalcitrant and endure against the aerobic digestion and oxidizing agents [10]. This significant resistance arises from azo dyes’ several benzene rings. Statistically 1%–20% of the entire production of dyes worldwide are wasted in the textile effluents [7,11] and adversely influence the metabolism of the human, ecosystem, and natural processes like eutrophication. The release of colored effluents into the aquatic media blocks sunlight and, thus, impedes the photosynthesis process of the algal and aquatic plants [12]. Moreover, it has been proven that azo dyes and their metabolites adversely affect humans and animals due to their toxicity, carcinogenicity, and mutagenicity [13].

Dyes could be classified based on their functional groups, color, and ionic charges upon dissolution in aqueous solutions [14]. The latter mode of classification based on the ionization of dye molecules, which intensively affects the efficiency of dye adsorption, is of higher importance. Accordingly, dyes can be classified to the ionic and non-ionic ones. The non-ionic dyes are divided to vat and disperse dyes, while the ionic ones are divided to cationic (basic) and anionic dyes (reactive, direct, and acidic) [5]. Table 1 tabulates the specific properties, application, and toxicity of each type of dye. As seen in the table, the listed dyes are mostly carcinogenic and must be treated effectively in the dye manufacturing factories to prevent their release into the environment.

## 2. Adsorption-Based Water Treatment

To resolve the significant challenge of water contamination by the discharge of dyes into water resources, various technologies have been scrutinized. Coagulation, flocculation, biodegradation, adsorption, ion-exchange, and advanced oxidation are the main techniques that have been investigated in this respect [8,9,15,16,17,18,19,20]. Among such approaches, adsorption is the most energy and cost effective one [3,21]. This technique enables removal of both groups of soluble and insoluble organic, inorganic, and biological water contaminants. As an example, for the sake of dye removal, the dye production industry traditionally employs commercial activated carbon thanks to its notable porosity and huge surface area (500–2000 m^2^·g^−1^). Nevertheless, this specific kind of adsorbent is produced at a high cost and, thus, is relatively expensive. Moreover, to regenerate the activated carbon, a high-pressure stream must be exerted that further raises the operation cost. Accordingly, such an expensive adsorption system has justified wide research in order to propose alternative dye adsorbents that are efficient and low cost [5]. In this regard, during the last decade, the agricultural waste derived inexpensive adsorbents, e.g., those originating from palm oil, spent rise biomass etc. have become interesting and widely researched [22]. Despite the promising applicability of these wastes for dye adsorption, they are mainly used as microparticles [23] whose limited accessible surface area drastically prolongs the time necessary to reach the maximum removal capacity of the pollutant. This characteristic is very challenging for industrial applications that demand a fast removal rate to address the ever-ascending pollutant volumes. Therefore, there is a crucial need to devise the sustainable adsorbents that are produced at a large scale at low cost and enable fast, efficient dye removal.

**Figure 2:**
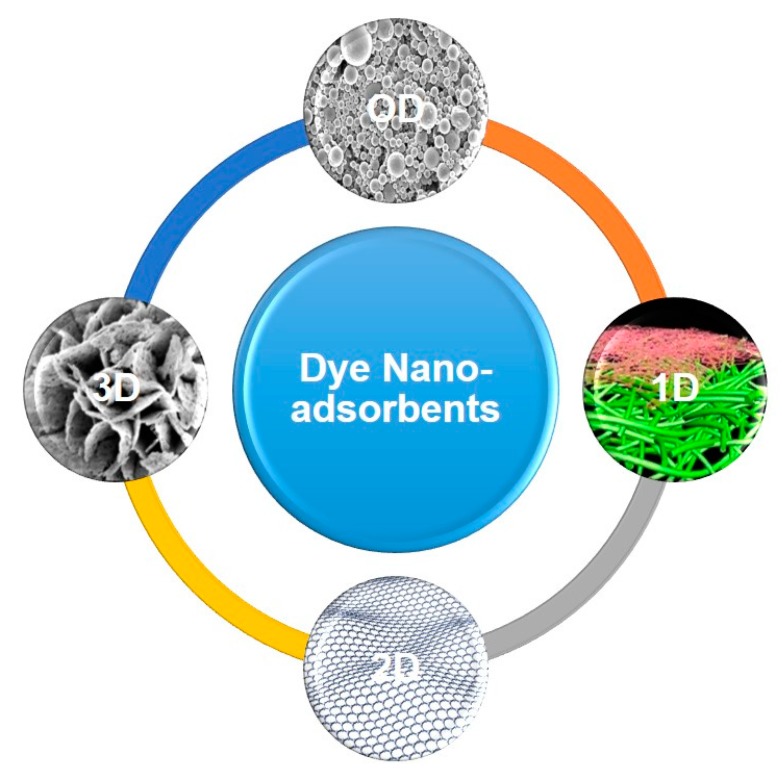
Various classes of the dye nano-adsorbents from the dimensionality standpoint. The used images for 0D, 1D, and 3D nano-adsorbents were reproduced with permission [25,26,27], respectively. The image related to the 2D nano-adsorbent was under a CC license.

To fulfil the mentioned requirements, during the past few decades, with evolution of nanotechnology, a diverse range of adsorbents has been developed whose dimensions do not exceed the nanoscale. Such nanomaterials offer a huge reactive surface area at a low mass and compared to activated carbon they can be synthesized at a lower cost and remove dyestuffs at a much lower quantity. With respect to dye removal, a variety of nano-adsorbents in different forms and dimensionalities (D) of nanoparticles (0D), nanofibers and nanotubes (1D), nanosheets (2D), and nanoflowers (3D) have been scrutinized. The 0D nano-adsorbents are those whose external dimensions lie in the nanoscale between 1 and 100 nm. Different types of nanoparticles belong to this category. 1D nano-adsorbents possess two nanoscale external dimensions and one microscale or even larger. Nanofibers, nanotubes, nanowires, and nanorods are classified under this dimensionality. Having only one nanoscale external dimension, nanosheets, thin films, nano-coatings, etc. are considered to be 2D nanomaterials. Lastly, 3D nano-adsorbents have no nanoscale external dimension while exhibiting internal nanoscale features. For instance, nanocomposites or assemblies of nanostructured materials can be considered 3D nanomaterials [24]. Figure 2 outlines the diverse classes of the dye nano-adsorbents in terms of dimensionality.

In the current review, the recently developed nano-adsorbents are introduced in a classified manner based on their dimensionality. In contrast to the relevant review papers slightly dealing with dye nano-adsorbents [28], or those published some years ago [3,5,29], naturally cannot reflect the current advancement in this field, in this draft, the main emphasis is put on presenting an updated introduction of such systems and providing a deeper, state-of-the-art insight into them. While some reviews solely discuss a specific class of nano-adsorbents such as nanoparticles [30,31,32], all families of nano-adsorbents including nanoparticles, nanotubes and nanofibers, nanosheets, and more complex 3D assemblies of basic 0–2D building blocks are taken into consideration. It is aimed to present a thorough classification based on dimensionality, composition, and operation mechanism of nano-adsorbents, which has been rarely seen in the reviews published in recent years [33,34,35].

## 3. Different Classes of Nanostructured Dye Adsorbents

### 3.1. 0D Nano-Adsorbents

Nanoparticles with an organic or inorganic origin have been extensively studied for the adsorption-based removal of dyes. Their large porosity, small size as well as huge surface area maximize their interaction with the dye molecules through their many pollutant binding sites and, thereby, their adsorption efficiency. Kinetically, the adsorption process mediated by the nanoparticles is fast. After saturation, they can also be optimally regenerated via different chemical processes [36]. In this case, some new studies related to the nanoparticle-based adsorption systems suggested for dye removal are briefly cited. The interested readers can refer to several excellent reviews published on this subject including References [3,5,37].

#### 3.1.1. Passive 0D Nano-Adsorbents

##### Biopolymer Derived 0D Nano-Adsorbents

In the past few decades, natural polymers and biopolymers have been paid large attention for the sake of adsorption based water treatment. For instance, polysaccharides including chitosan and its derivatives have shown a promising applicability for adsorption and, thus, removal of organic and inorganic contaminants from water [38,39]. Compared to the classic adsorbents such as activated carbon, chitosan can be produced in a less costly manner while offering a high adsorption efficiency thanks to a large number of amino and hydroxyl functional groups, which enables removal of a variety of water contaminants [40,41]. Shajahan et al. [42] synthesized fungal chitosan nanoparticles (2–30 nm in diameter) from *Cunninghamella echinulata* (Thaxter) and characterized their potential for dye (remazol brilliant blue (RBB), methyl orange (MO), disperse red 13(DR), napthol blue black (NBB), and Chicago sky blue 6B (CSB)) adsorption. The anionic dyes can readily adsorb onto the chitosan nanoparticles thanks to an electrostatic interaction with the positively charged amino groups of chitosan. Dhananasekaran et al. [43] synthesized α-chitin nanoparticles as small as <50 nm from *Penaeus monodon* shell waste and challenged them with respect to dye adsorption efficiency. For the tests, they employed Methylene blue (MB), Bromophenol Blue (BPB), and Coomassie Brilliant Blue (CBB) as dye models. For the mentioned dyes, an adsorption efficiency up to 95.96% and 99%, respectively, depending on the adsorbent concentration, was recorded. This large adsorption efficiency is attributed to the physical adsorption of the dyestuff to the nanoparticles.

##### Synthetic Polymer Derived 0D Nano-Adsorbents

Other than the natural organic nanoparticles, the synthetic ones have also been studied for dye adsorption. For instance, polyaniline nanoparticles were synthesized by Saad et al. [44] for adsorption-based removal of the Crystal Violet (CV) dye. The process was facilitated by involvement of ultrasonication that could maximize mass transfer. The nanoparticles were efficient in the removal of 94.29% dye from water. Liu et al. [45] developed Davankov-type hyper-crosslinked-polymer (HCP) nanoparticles that could be used for adsorption of the benzene-ring-containing dyes. The nanoparticles were synthesized based on a poly(DVB-co-VBC) precursor and were hydrophobic and stable. By adding the HCP nanoparticles to aqueous solutions containing MB, nigrosine, and acid orange (AO), a high adsorption efficiency of 96%, 97%, and 94%, respectively, was achieved. The dye molecules were mainly adsorbed onto the surface of the nanoparticles rather than diffused into the surface porosity. A benzene ring containing dye possessing a highly conjugated structure can readily and promptly adsorb onto the highly aromatic HCP nanoparticles. However, in the case of the dye models such as Prussian Blue that are free of benzene rings, this performance is not observed.

To address the need for adsorbent materials with a huge surface area, plenty of functional groups and open porosity, maximizing the adsorption efficiency and rate, Ou et al. [46] synthesized a rich amine porous organic polymer (RAPOP) via the Schiff base reaction. The nanoparticles made of such a polymer possess a large number of micro/mesopores formed via the polymerization reaction between melamine and terephthalaldehyde monomers. The RAPOP-based adsorbent systems show a myriad of promising features such as an extensive specific surface area (368.05 m^2^·g^−1^), large porosity (0.651 cm^3^·g^−1^), optimum physicochemical stability, insignificant density, and, most importantly, an extraordinary functionality due to the presence of many exposed functional groups. This nano-adsorbent is able to effectively remove 454.545 mg·g^−1^ MO from water favorably in an acidic condition. The adsorption equilibrium time was 4 h at room temperature.

##### Inorganic 0D Nano-Adsorbents

Inorganic nanoparticles have also been widely studied for the sake of dye removal. For instance, Li et al. [47] amorphized transitional metal oxide nanoparticles of Fe, Co, and Ni, i.e., Fe_2_O_3_, CoO, and NiO for the adsorption of MB through a novel treatment based on laser irradiation in liquid. The maximum adsorption capacity for the NiO amorphous nanoparticles was reported as much as 10584.6 mg·g^−1^. This high capacity is attributed to increase of the exposed surface area after the amorphization process, and intensive ionic bonding between M^2+^ in M (OH^+^) and O^2−^ in the sulfonic groups of MB. Meng et al. [48] synthesized Cr-doped ZnO (with the optimal Cr/Zn ratio of 6%) nanoparticles that could remarkably remove MO molecules from water. For this adsorbent system, a high adsorption capacity of 310.56 mg·g^−1^ has been reported that is achieved in a short time. There are many other inorganic nanoparticle-based adsorbents including MnO_2_ [49], ZnS:Cu [50], Cu_2_O [51], and ZnO:Cr [52] that have been coupled with activated carbon, which engender a highly efficient dye removal. In such systems, activated carbon acts as a support for the nanoparticles and also offers further reactive binding sites such as OH, COOH, C=O, and amide groups that, alongside the nanoparticle, cooperatively raise the adsorption efficiency [50].

**Figure 3:**
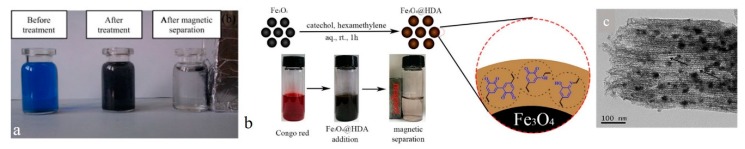
(**a**) Camera images demonstrate the various stages of water decolorization by the magnetic nanoparticles. Reproduced with permission [55]. Copyright 2016, Elsevier. (**b**) The schematic shows the synthesis procedure of the amino-coated Fe_3_O_4_ nanoparticles and their adsorption efficiency for the CR dye model. Reproduced with permission [56]. Copyright 2018, Elsevier. (**c**) TEM image implies embedment of Fe/Ni magnetic nanoparticles within the mesoporous carbon. Reproduced with permission [57]. Copyright 2015, Elsevier.

One major challenge related to the adsorbent nanoparticles is their recovery after application due to their small size. Traditionally, separation of the nanoparticle adsorbents from treated water takes place by filtration and sedimentation techniques where the small dimension of such adsorbents potentially gives rise to blockage of the filters or release of the nanoparticles and, thus, a secondary pollution [53]. In addition, the aggregation tendency of nanoparticles is notably high and this could engender loss of adsorption efficiency. For such bottlenecks, they are coupled with materials in larger dimensionalities such as activated carbon and nanofibers [6] or made of (or combined with) magnetic materials that enable their collection. The adsorption processes on the basis of magnetic nanoparticles are simple, fast, inexpensive, and easily integrated into automation methods [54]. Dalvand et al. [55] synthesized Fe_3_O_4_ magnetic nanoparticles surface-treated with L-arginine (Fe_3_O_4_@L-arginine). They benefited from this material in the removal of Reactive Blue 19 azo dye from wastewater streams. Under the most desired circumstances, i.e., a primary dye concentration of 50 mg·L^−1^, an adsorbent dose of 0.74 g·L^−1^, and pH 3, Fe_3_O_4_@L-arginine nanoparticles could show a high removal efficiency of 96.34%. As shown in Figure 3a, the dye holding Fe_3_O_4_@L-arginine nanoparticles can be readily and promptly (less than 20 s) separated from water by applying an external magnet. Amination is another surface treatment strategy for the Fe_3_O_4_ nanoparticles and, thereby, for dye adsorption. Dai et al. [56] performed the mussel-inspired polymerization to synthesize amino-coated Fe_3_O_4_ nanoparticles (Figure 3b). To do this, they simply immersed Fe_3_O_4_ nanoparticles into an aqueous solution of catechol and hexanediamine (HDA). The adsorption capacity of the system for congo red (CR) was 97.3 mg·g^−1^ and 80% of the equilibrium adsorption amount was fulfilled within 200 min. For such a promising adsorption performance, the following grounds could be considered: (1) The electrostatic interaction between the charged amino and/or carboxyl groups from HDA and lysine present on the surface treated nanoparticles and dye molecules, (2) the π−π stacking interaction between the aromatic rings in the catecholic coating of the nanoparticles and those in the dye molecules, and (3) the hydrogen bonding between the phenolic hydroxyl groups in the catecholic coating and dye molecules.

The adsorbents combining magnetic nanoparticles and mesoporous carbon have also been reported [57]. Replacing activated carbons and zeolites whose microporosity could be problematic with respect to adsorption of large dye molecules, ordered mesoporous carbon materials have shown promising applicability for dye removal. This class of adsorbents show optimum characteristics including regular mesoporosity, a huge specific surface area, and remarkable pore volume, which are desirable for the dye adsorption purpose [57,58]. To enhance the recovery of such adsorbents through a magnetic separation method, Fe-based, Ni-based, and Co-based nanoparticles have been added to them [59,60]. While this approach enables the magnetic recovery, it can also lead to blockage of the pores and lead to declining of the mass transfer, which lowers adsorption efficiency [61]. Accordingly, magnetic nanoparticles are preferred to be embedded within the mesoporous carbon adsorbents. In this regard, Liu et al. [57] developed magnetic Fe/Ni nanoparticles embedded bimodal mesoporous carbon for adsorption of MB cationic dye and MO anionic dye models (Figure 3c). The adsorption capacity was affected by pH. While alkaline pH was more desirable for adsorption of MB, MO molecules were adsorbed to the adsorbent under acidic pH. Kinetically, the adsorption followed a pseudo-second-order model and showed a 3-stage intraparticle diffusion mode. The obtained adsorption data complied well with the Langmuir model, and implied a maximum adsorption capacity for MB and MO as much as 959.5 mg·g^−1^ and 849.3 mg·g^−1^, respectively. Thermodynamically, the adsorption process was spontaneous and endothermic.

##### Nanocomposite 0D Nano-Adsorbents

**Figure 4:**
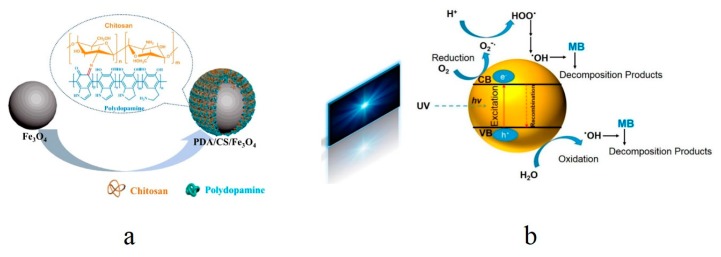
(**a**) A nanocomposite adsorbent comprising polydopamine/chitosan/magnetite nanoparticle core. Reproduced with permission [64]. Copyright 2016, Elsevier. (**b**) The schematic illustrates the photocatalysis process of TiO_2_ engendering the dissociation of the neighboring MB molecules. Reproduced with permission [67].

In the last few years, there has been a fast, extensive progress in the field of nanocomposites. Novel, advanced nanocomposites in different dimensionalities (0D–3D) have emerged [62,63]. The nanocomposite nanoparticles comprising organic and inorganic components are another class of the nanoparticulate dye adsorbents. In this regard, Tanhaei et al. [10] synthesized a novel chitosan/Al_2_O_3_/magnetic iron oxide nanoparticle composite that could adsorb MO from water. In general, the magnetic core-chitosan shell nanoparticles have shown optimum applicability for water treatment. While magnetite as the magnetic core is able to offer a superparamagnetic effect and is synthesized in an inexpensive, simple manner, it is vulnerable to acidic conditions and could lose its magnetic intensity. Thus, coating of it with an inert biomaterial such as chitosan can preserve its magnetism for a long time. Moreover, Al_2_O_3_ raises the chemical stability of nanoparticles, their oxidation resistance, and, possibly, the system’s selectivity for ion uptake. The adsorption isotherm complied well with the Langmuir model and a high MO adsorption capacity of 1.27 mmol·g^−1^, i.e., 417 mg·g^−1^ at 25 °C was recorded. The adsorbent was also reusable when treated with HCl (0.1 M). In another study, Wang et al. [64] developed a nanocomposite nanoparticle adsorbent for dye removal that comprised a magnetic Fe_3_O_4_ nanoparticle core surface decorated with a hybrid coating of chitosan and polydopamine (PDA) (Figure 4a). The nanoparticle confers a large surface area for adsorption while the functional coating offers many possibilities for an interaction with the dye molecules. As a result, the adsorbent was able to remove 204 mg·g^−1^ and 61 mg·g^−1^, i.e., 96.9% and 92.5% of MB and MG, respectively. A similar system based on the Fe_3_O_4_ magnetic nanoparticle as core coated with a co-deposited layer of PDA and polyethylenimine (PEI) has been reported by Wang et al. [65]. The as-developed adsorbent comprises an ultrathin coating layer (3 nm) and desirably resists against intense alkaline solutions (0.1 M NaOH, pH = 13). Moreover, it efficiently and promptly removes (>95% in just five minutes) anionic dyes from dye mixtures in a selective manner. The high removal efficiency (>90%) persists after 10 cycles. An inorganic nanocomposite nanoparticle of CuO/ZnO was synthesized on a bio-template of the eggshell membrane [66]. The eggshell membrane is composed of protein (collagens and glycoproteins) fibers that are arranged as interwoven fibrous structures. Such a unique porous structure possessing a huge density of multiple functional groups, e.g., COOH, NH_2_, OH etc. enables strong adsorption and chelation of metal ions and, thereby, synthesis of respective nanomaterials, as done in this study for the CuO/ZnO nanocomposite. This composite/biohybrid nanostructure allowed for an excellent adsorption capacity for CR dye by as much as 775 mg·g^−1^.

#### 3.1.2. Active 0D Nano-Adsorbents

The previously mentioned dye removal processes such as adsorption or coagulation only gather the dye molecules by transforming them to other phases but do not entirely “eliminate” or “decompose” them. This matter could be problematic because disposal of the dye-related sludge is challenging and the adsorbent is hardly reusable [68]. For such reasons, the adsorption process could be complemented by the degradation processes such as photocatalysis, sonocatalysis, and reductive degradation that allow decomposition of dye to nontoxic metabolites.

In general, various classes of advanced oxidation processes (AOPs), which enable release of hydroxyl radicals (OH•), have proven efficient in decolorization of textile effluents. Possessing unpaired electrons, OH• is extremely reactive and can oxidize resistant organics [69]. Thanks to the availability of inexpensive, efficient photocatalysts, photocatalysis is one of the most studied AOP processes and is recognized as an efficient dissociation process for organic pollutants, such as dyes and pesticides. This kind of contaminant is readily decomposed in aqueous solutions induced by the photocatalytic activity of various semiconductor metal oxide nanoparticles.

There is a variety of photocatalysts introduced for water treatment such as CdS [70], SnO_2_ [71], MgO [72], WO_3_ [73], SiO_2_ [74], ZnO [75,76], Fe_2_O_3_ [77], CuO [2], and TiO_2_ [67,78]. Thanks to the size-governed physicochemical properties, superior activity, endurance, and inexpensiveness, these nanomaterials have been extensively studied for the sake of photocatalytic degradation of dye molecules [79]. Among the above mentioned photocatalysts, TiO_2_ is the most studied one due to its remarkable efficiency, low cost, physicochemical endurance, and abundance [80,81]. The UV-irradiation of TiO_2_ nanoparticles provokes the valence band (VB) electrons and shifts them to the conduction band (CB), which leads to the formation of energized “holes” in the VB (Figure 4b) [80,82,83]. Subsequently, the generated free electrons react with oxygen, which gives rise to the formation of superoxide radical anions (O_2_^∙−^). Meanwhile, the energized holes oxidize water (H_2_O) or hydroxyl ion (OH^−^), whereby producing hydroxyl radicals (•OH) that can dissociate the neighboring dye molecules optimally [67]. Other than dye decomposition, water oxidation by photocatalytic nanoparticles, e.g., TiO_2_ [84], ZnO [85], and cobalt oxide-based nanoparticles [86] has also gained wide attention for the sake of energy production and has been employed for artificial photosynthesis and photoelectrochemical water-splitting systems. The water oxidation process, which is also called as the oxygen evolution reaction (OER), takes place in parallel with the hydrogen evolution reaction (HER) during the electrolysis of H_2_O [87], which leads to the generation of renewable H_2_ [85].

To offer an energy efficient version of photocatalysis that is also eco-friendly, visible light-induced photocatalysis has drawn extensive attention in recent years. This interest arises from this reality that UV light accounts for a negligible part (≈ 5%) of the sun’s energy in contrast to the visible part (43%) [88]. Accordingly, for the sake of energy efficient photocatalysis, there is a need for suitable visible-light photocatalysts that enable the degradation of pollutants solely under the solar energy. For instance, CdS is one of the most important II–VI semiconductors with a large direct band gap of 2.42 eV. This band gap enables easy generation of photoelectrons and holes even under visible light irradiation [88]. In this context, a diverse range of hierarchical photocatalysts such as Bi_2_MoO_6_, Ag_3_VO_4_/Bi_2_O_2_CO_3_, Ag_2_O/BiOCOOH, and Ta_3_N_5_/Bi_2_MoO_6_ have been introduced [89,90,91,92,93]. Another strategy to lower the band gap of a photocatalyst is doping with various elements. For instance, the photodecomposition efficiency of ZnO nanoparticles rises when doped with metal ions of Mn^2+^, Mg^2+^, Cd^2+^, Cr^2+^, Fe^3+^, Cu^2+^, and Ag^+^ [48,94,95,96,97,98,99]. The ZnO photocatalyst is able to absorb both UV and visible light and shows a large band gap energy of 3.2 eV, tailorable electrical conductivity, and a high excitation energy of 60 meV [100]. Therefore, ZnO nanoparticles are regarded as promising potential photocatalysts for removing toxic pollutants such as dyes. Jena et al. [101] synthesized Fe^3+^-doped ZnO nanoparticles for the photodegradation of MO dyes in water. In this study, it was found that an increase of the dopant concentration leads to shrinkage of the band gap so that, when the Fe^3+^ percentage rises to 5%, the bandgap decreases to 2.61 eV.

MgO has also been studied as a photocatalyst for dye removal due to its excellent chemical, mechanical, optical and electrical characteristics, large energy band gap, low cost, robustness, and safety. Despite such merits, the high required energy, and the necessity of application of ultraviolet C (UVC) to achieve optimum photocatalytic results could be somewhat hindering. To address this bottleneck, Jorfi et al. [72] coupled a coagulation pretreatment process (using AlCl_3_ coagulant) to the photocatalytic one assisted with ultraviolet A (UVA) irradiation to degrade AR 73 dye. When challenged by a real textile wastewater, the mentioned combined process enabled notable total chemical oxygen demand (COD) and total organic carbon (TOC) removal of 98.3% and 86.9%, respectively, after 5 h.

Despite wide employment of photocatalysis, it is by no means energy and time efficient [68]. Sonocatalysis is also considered a supplementary treatment for adsorption and can effectively degrade the adsorbed dye molecules. The underlying mechanism of sonocatalysis is acoustic cavitation. In such a process, bubbles form, expand, and collapse in the solution. When the bubbles are exploded, localized hot spots with a very high temperature and pressure form, and, thereby, thermally dissociate the dissolved oxygen and water molecules and generate extremely reactive radicals of •OH, •H, and •O, which are able to decompose dye molecules in water. By including a nanocatalyst, the sonocatalytic degradation efficiency is optimized because: (i) the presence of the nanocatalysts offers extra nuclei for the generation of the cavitation bubbles, (ii) the ultrasound irradiation facilitates the mass transfer of dye (or any other organic pollutants) between the aqueous and solid phases, (iii) the ultrasound irradiation crushes the formed aggregates and, thus, raises the exposed surface area, and (iv) the catalyst is provoked by ultrasound-governed luminescence, which further generates •OH. In this regard, Bansal et al. [68] used ZrO_2_ nanoparticles to sonocatalytically remove cationic (Victoria Blue) and anionic (Direct Red 81) dyes. Compared to the photocatalytically excited nanoparticles, a much larger degradation efficiency was recorded for the nanoparticles treated sonocatalytically for either the dye pollutant models.

**Figure 5:**
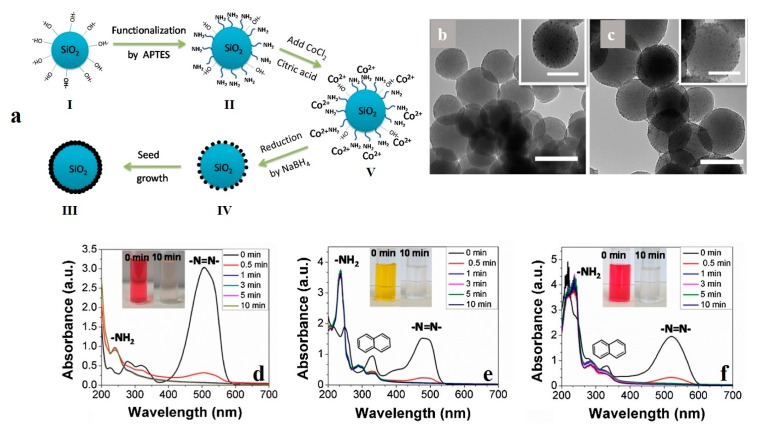
(**a**) The synthesis procedure for SiO_2_-Co core-shell nanoparticles. TEM images imply the SiO_2_-Co core-shell nanoparticles prepared from two different Co^2+^ precursor amounts of (**b**) 1 mM and (**c**) 2 mM Co^2+^ (Scale bar in the main image and inset represents 100 nm and 50 nm, respectively). UV/Vis spectra indicate the model dye degradation by the core-shell nanoparticles over time (note that the solutions’ pH was acidic (pH 2.5) and the initial dye concentration was 0.076 mM) (**d**) Methyl Orange. (**e**) Orange G. (**f**) Amaranth. Reproduced with permission [103]. Copyright 2016, Elsevier.

With respect to reductive degradation, Sha et al. [102] developed hollow cobalt nanoparticles that could remove MO from water quickly. The cobalt nanoparticles, synthesized through a galvanic replacement reaction using aluminum nanoparticle templates, were able to reduce MO to amine compounds in a few minutes. Precisely speaking, for a primary MO concentration of 100 mg·L^−1^ (pH = 2.5) and using Co nanoparticle with the dosage of 0.5 g·L^−1^, the dye degradation efficiency could be as high as 99% in only 4 min. In this process, the degradation constant rate was around 2.444 min^−1^, which could be regarded as the highest reported rate. The degradation performance of the hollow cobalt nanoparticles prevailed notably over solid Co and Ni nanoparticle counterparts. In a relevant study, Zhang et al. [103] synthesized SiO_2_-Co core shell nanoparticles for reductive degradation of azo (MO) dyes. As shown in Figure 5a, they functionalized the surface of SiO_2_ nanoparticles by using (3-Aminopropyl) triethoxysilane (APTES, 99%), providing amine terminal groups. Subsequently, the surface-treated nanoparticles were subjected to cobalt chloride. The cobalt cations were, therefore, reduced by the chelating amine and hydroxyl groups emerged on the surface. This process eventually led to the formation of a Co shell. Figure 5b,c shows the TEM images related to the SiO_2_-Co core-shell nanoparticles synthesized with two Co precursor concentrations of 1 and 2 mM. While, at the lower concentration, Co seeds from on the surface of the nanoparticles, at the larger amount of the precursor, the Co domains grow and shape a coherent shell around the SiO_2_ nanocores. The as-developed Co/SiO_2_ nanoparticles show optimum degradation efficiency for azo dyes including MO, orange G, and amaranth (Figure 5d–f).

Despite expanding application of metal oxide nanoparticles in water treatment, they impose several adverse effects on the health of living organisms including human. The toxic effect of the metal oxide nanoparticles have been investigated and the cytotoxic potential of them has been proven by several research teams [104]. For instance, iron oxide nanoparticles (e.g., Fe_3_O_4_) that are widely employed for recovery of the nano-adsorbents have shown toxicity effect in different in vitro and in vivo studies [105]. TiO_2_ is another metal oxide that, as a nanoparticle, is being largely used for photodegradation of dyes. It has been demonstrated that the intragastrically administered TiO_2_ nanoparticles (2.5 to 10 mg·kg^−1^ body weight) provoke spleen damage and immune dysfunction in mice after three months [106]. Moreover, stress induced alterations in the expression of genes, cell proliferation, apoptosis, metabolic processes, and oxidative stress have been recorded. ZnO nanoparticles have shown to be efficient antimicrobial and anticancer agents. Yet, their toxicity against human cell lines needs further investigations. ZrO_2_ nanoparticles that have been used with respect to the sonocatalytic degradation of dyes could be also applied as a constituting material of dental and orthopedic materials. The in vivo studies on the ZrO_2_ containing yttrium oxide for two years have shown no particular toxicity effect [107]. According to the performed studies, the nanotoxicity of metal oxide nanoparticles could be attributed to (i) the likely release of (toxic) ions from the nanoparticles and (ii) the oxidative stress engendered by the nanoparticle’s intrinsic characteristic such as morphology, surface charge, size, and surface chemistry or composition [108]. In this regard, additional studies are necessary to help comprehend the involved mechanisms. The toxicity protocols should be standardized, and the nanoparticle toxicity should be studied over the long term. Most importantly, the fate of this kind of nanoparticle in human tissue and, in the environment, should be clarified through extensive tests.

### 3.2. 1D Nano-Adsorbents

1D nanostructures including nanotubes, nanoneedles, nanofilaments, and, ultimately, nanofibers have been also considered for dye adsorption and degradation. Such nanostructures offer a large aspect ratio and extensive surface area thus optimum capability for adsorption of different dyes physically and/or chemically. This opportunity is enhanced when the surface is further functionalized and the available porosity is promoted.

#### 3.2.1. 1D Carbonaceous Nano-Adsorbents

Since their discovery in 1991, hollow carbon nanotubes (CNTs) whose diameter lies in the nanoscale range have been widely studied across the world for diverse utilities in electronics, hydrogen storage, catalysis, and drug delivery [109]. CNTs show an extensive specific surface area, small size, and remarkable chemical and thermal stability, thus offering amazing potential for adsorption of dye molecules as well [110]. However, they are hardly dispersible in aqueous systems. The lack of functionality and density of functional groups challenge their adsorption efficiency. Accordingly, development of the functionalized CNTs able to adsorb a diverse range of cationic or anionic dyes is of the utmost importance. For instance, thiolated multi-walled carbon nanotubes (MWCNTs) have shown promising adsorption capacity for cationic MB [111]. A more sophisticated functionalized CNT-based adsorbent was developed by Xie et al. [109] through polymer grafting. They suggested an approach based on a combination of mussel chemistry using PDA and Single-Electron Transfer Living Radical Polymerization (SET-LRP), which engenders poly(sodium-*p*-styrene sulfonate) (PSPS) functionalized MWCNT. The CNT-PDA is initially synthesized through the self-polymerization of dopamine, which leads to coverage of the CNT’s surface with PDA under an alkaline condition. Next, the SET-LRP initiator (CNT-PD-Br) is made through amidation and esterification reactions of CNT-PDA and 2-bromo-2-methylpropionyl bromide. Eventually, by polymerization of the sodium-*p*-styrene sulfonate hydrate (PSPSH) monomers, the CNT-PDA-PSPSH system is realized. The as-developed adsorbent showed a high MB adsorption capacity of 160 mg·g^−1^.

Other than polymer grafting, biomaterial grafting has been also employed to raise functionality thus adsorption capacity of CNTs. In this regard, chitosan (CS) can be covalently loaded onto the CNT and enhance its functionality as well as mechanical stability. Mahmoodian et al. [112] synthesized poly(2-hydroxyethyl methacrylate) polyHEMA–CS-f-MWCNT and investigated its potential for adsorption of MO. According to their measurements, the adsorbent was able to remove 306.6 mg·g^−1^ MO from water, mainly due to the electrostatic interaction between the amine groups of the graft (particularly the chitosan section) and anionic dye molecules that is intensified under acidic pHs.

To recover such small nano-adsorbents, the solution containing the CNTs need to be filtered and/or centrifuged. Such methods are lengthy and there is the possibility of fouling of the filters by the CNTs and, thereby, waste of the adsorbent. Similar to the nanoparticulate adsorbents, magnetic recovery could be an optimum strategy to preserve the CNT adsorbents. Fe_3_O_4_ nanoparticles show optimum chemical stability, monodisperse particle size, and biocompatibility, and can act as a complementary unit alongside the CNTs, which allows their magnetic recovery. Sun et al. [113] employed Fe_3_O_4_/CNTs for the adsorption-based removal of sudan I-IV dyes from water. In addition, Duman et al. [110] developed magnetic oxidized multi-walled carbon nanotube (OMWCNT)-Fe_3_O_4_ and OMWCNT-κ-carrageenan-Fe_3_O_4_ nanocomposites and employed them for the adsorption of MB. κ-carrageenan is a sulfated anionic polysaccharide extracted from seaweed and is highly hydroxylated. Therefore, it is negatively charged. Moreover, chemical oxidation of CNTs confers them with a large number of carboxyl groups that enable esterification with the OH groups and, thereby, linkage with the natural polymer of κ-carrageenan. This system shows a very optimum adsorption efficiency for cationic dyes such as MB due to hydrogen bonding, π–π stacking (between the aromatic backbone of MB and the hexagonal structure of the OMWCNT), and electrostatic interactions. A nanohybrid adsorbent comprising magnetic Ni nanoparticles and porous carbon-CNTs has also been developed based on a Ni/Zn-MOF precursor, which is later carbonized. The pyrolysis-induced removal of Zn brings about a high porosity and a large surface area for the adsorption process. This system offers not only optimum adsorption efficiency for a variety of dye models including MG (898 mg·g^−1^), CR (818 mg·g^−1^), Rh B (395 mg·g^−1^), MB (312 mg·g^−1^), and MO (271 mg·g^−1^), but also the possibility of magnetic separation of the adsorbent elements at the end of the purification process [114]. The Ni/CNT system shows an extensive surface area of 999 m^2^·g^−1^ and high porosity of 0.86 cm^3^·g^−1^ that facilitate adsorption of the dye molecules. Despite the dye removal efficiency of the CNTs, their large-scale application is restricted and largely dependent on the economical grounds and needs to be justified with cost reduction, which is currently challenging. Additionally, the unprocessed CNT might contain metal catalysts and impurities that can potentially induce a toxicity effect. The small size of the CNTs also facilitate their entry into the living systems. In addition, their extensive surface area, surface charge, and aggregation tendency would lead to pro-inflammatory effects (particularly in lung tissue). The translocation of the CNTs from one organ to other vital ones is imaginable. This process can influence blood, skin, gut, brain, liver, etc. and damage them. The adverse effect of such nanomaterials arises from their shape, size, surface area, and charge, which cause a notable chemical and biological activity, thereby, producing a large amount of reactive oxygen species (ROS). The generation of ROS and free radicals could engender oxidative stress, inflammation, and, thereby, damages to proteins, membranes, and DNA [115]. Regarding the health and environmental implications of the CNTs, the interested reader is referred to Reference [116]. Accordingly, any further research on the CNT for water treatment is notably governed by production cost reduction and minimizing the presence and dose of the likely toxic elements.

**Figure 6:**
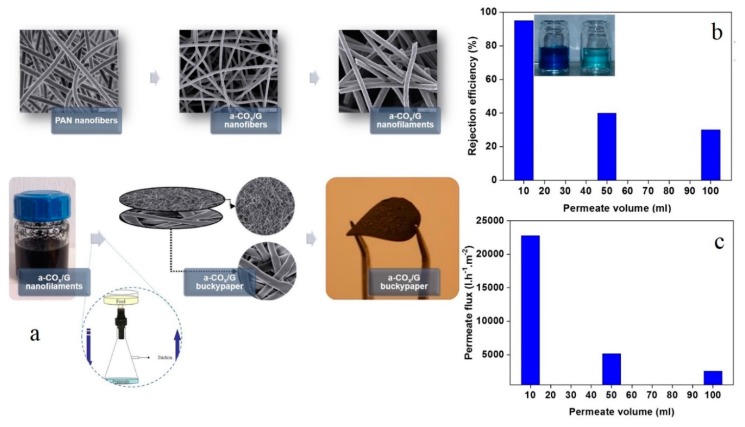
(**a**) The schematic illustrates the construction procedure of the a-CO*_x_*/G adsorbent membrane (the scale bars represent 2 µm and 1 µm for the first two and third images, respectively). (**b**) MB removal efficiency of the a-CO*_x_*/G adsorbent (the inset shows the feed and permeate samples, respectively) and (**c**) the solution flux of the a-CO*_x_*/G adsorbent. Reproduced with permission [117]. Copyright 2018, John Wiley and Sons.

As a novel class of pure 1D carbon nano-adsorbents, Homaeigohar et al. [117] synthesized amphiphilic oxygenated amorphous carbon/graphite (a-CO*_x_*/G) hybrid nanofilaments (Figure 6a). The co-existence of the nanofilaments’ polar and nonpolar regions, corresponding to a-CO_x_ and G, respectively, brings about amphiphilicity, thus capability of interaction with the neighbouring polar/nonpolar substances [118,119]. Specifically, induced by electrostatic and π–π interactions, the nanofilaments favorably capture MB dye from water. The a-CO*_x_*/G hybrid nanofilaments allowed for dynamic separation of 95% MB from water while maintaining a high water permeability of 23 kL·h^−1^·m^−2^, as represented in Figure 6b,c. Despite a highly promising primary adsorption efficiency, as long as the dynamic separation went on the removal efficiency and water permeability dropped, presumably due to the occupation of the binding sites by the MB molecules and, thereby, blockage of water passages.

#### 3.2.2. 1D Biopolymer Nano-Adsorbents

Cellulose is the most available biopolymer and a recyclable bioresource, which promises for construction of many advanced functional materials [120]. The cellulosic fiber is a long-chain polysaccharide, which comprises β-d-glucopyranose units. There are three OH groups on each hydroglucose that could act as active sites for adsorption of dye molecules. Cellulose includes amorphous and dense crystalline regions arranged by hydrogen bonds and van der Waals forces. By exposure of cellulose to a properly adjusted series of mechanical, chemical, and/or enzymatic processes, single, separated nanofibers, i.e., cellulose nanocrystals (CNCs) are resulted by dissociation of the amorphous regions [121].

The cellulose nanocrystals have shown applicability in the removal of various dye pollutants, particularly rhodamine (Rh) dyes. This wide utility originates from the optimum interaction between nanocellulose and dye through hydrogen bonding and electrostatic interaction. With respect to the latter possibility, the negatively charged (O^−^) structure of nanocellulose enables a strong binding with cationic dyes [122]. Functionalization of cellulose has shown efficient in the extension of applicability of cellulose nano-adsorbents. The amine-functionalized cellulose nano-whiskers have also been applied in removing anionic dyes. The functionalization by ethylenediamine engenders many primary amine groups onto the nano-whiskers that enable adsorption of dye acid red GR. In fact, the electrostatic interaction between the protonated amine groups and the anionic dye is responsible for the optimum dye removal efficiency of such an adsorbent. Accordingly, the adsorbent succeeded to adsorb 555.6 mg·g^−1^ of acid red GR. Kinetically, the adsorption behavior followed a pseudo-second order model and, thus, implied a chemisorption-based adsorption [123]. With respect to the functionalized cellulose derived dye adsorbents, another system, i.e., polyvinylamine-functionalized cellulose nanocomposite, has shown a high efficiency in removing anionic dyes such as reactive light yellow K-4G, congo red 4BS, and acid red GR [124].

**Figure 7:**
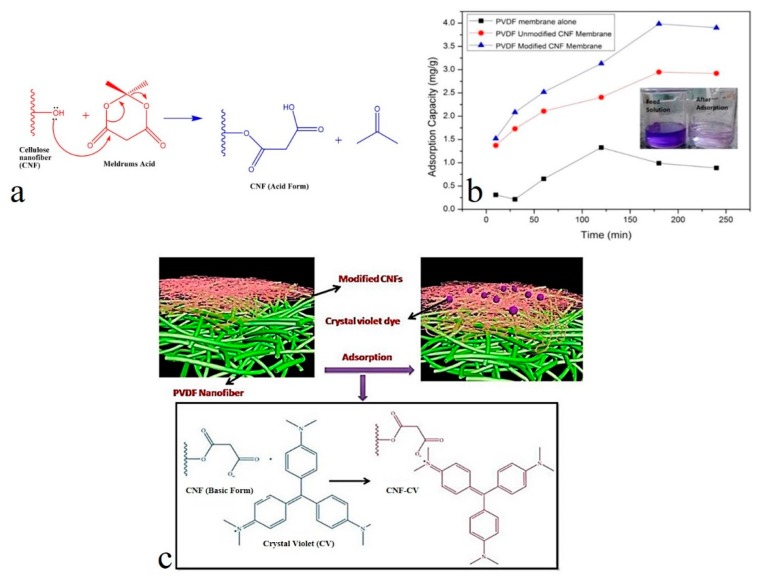
(**a**) The functionalization reaction that engenders the Meldrum’s acid modified cellulose nanofiber. (**b**) Time-dependent CV adsorption efficiency of the Meldrum’s acid modified cellulose nanofiber versus that of the control samples including the PVDF nanofibers and the neat cellulose nanofibers + PVDF nanofibers. (**c**) The upper row schematically illustrates the CV adsorption process of the Meldrum’s acid modified cellulose nanofiber placed on a PVDF support layer while the lower row showcases the underlying adsorption mechanism. Reproduced with permission [26]. Copyright 2017, American Chemical Society.

Among the biopolymer derived 1D nanomaterials, cellulose nanofibers can potentially offer amazing adsorption capacity thanks to their low cost, large density of hydroxyl groups, environmentally-friendly being, and abundance. The hydroxyl groups available on the surface also enable further functionalization and attachment of ligands and functional agents that maximize adsorption capacity of the nanofibers [26]. In this regard, cellulose nanomaterials have been surface-treated through (2,2,6,6-tetramethylpiperidin-1-yl)oxyl (TEMPO) oxidation [120,125,126] and by using a pyridine solvent [121]. While using TEMPO and pyridine imposes environmental and economical burden and toxicity, the non-solvent-based surface treatment of cellulose is gaining wide attention. In this regard, Gopakumar et al. [26] applied Meldrum’s acid (2,2-dimethyl-1,3-dioxane-4,6-dione) as an esterification agent to functionalize cellulose nanofibers in a green manner (Figure 7a). Accordingly, the surface-treated cellulose nanofibers acquire a negative surface charge that allows them to remove the cationic CV molecules from water. By loading the modified cellulose nanofiber layer on a polyvinylidene fluoride (PVDF) electrospun support layer, the assembly shows a dye adsorption efficiency of 3.984 mg·g^−1^, which is superior to that of its unmodified counterpart and the bare PVDF support layer (Figure 7b). Such an optimum adsorption efficiency, as seen in Figure 7c, is attributed to the electrostatic interaction between the negatively charged carboxyl groups and the positively charged CV molecules.

#### 3.2.3. 1D Inorganic Nano-Adsorbents

**Figure 8:**
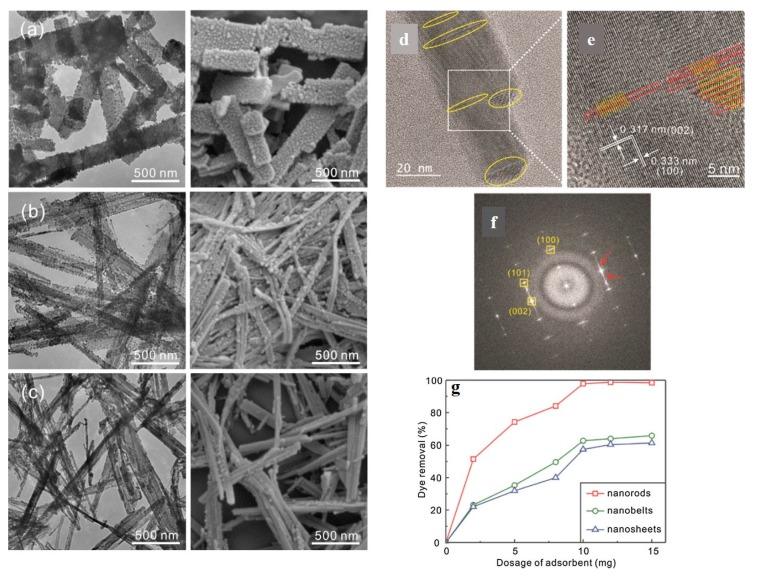
(**a**–**c**) TEM (**left**) and SEM (**right**) images imply the morphology of the Cu exchanged ZnS nanosheets, nanobelts, and nanorods, respectively. HRTEM images (**d,e**) and FFT pattern (**f**) of a Cu-exchanged ZnS nanorod. In these images (**d** and **e**), the yellow marks and lines represent the stacking faults and planar defects, respectively. In addition, the red lines (**e**) and arrows (**f**) mark the lattice orientations and defects, respectively. (**g**) RhB removal efficiency of the Cu-exchanged ZnS nano-adsorbents in different morphologies varies depending on the adsorbent concentration. Reproduced with permission [127]. Copyright 2017, Elsevier.

Despite the promising capabilities of CNTs in physical adsorption of dye molecules through the van der Waals forces, hydrogen bonding, and electrostatic interaction, they are poor in terms of dispersibility in the wastewater systems, are regenerated in a costly manner, and are non-selective toward ionic contaminants [127]. Such shortcomings have driven new studies to replace the CNTs with other materials including the inorganic ones. In this regard, the largely available, eco-friendly metal sulfides e.g., ZnS synthesized as 1D nanostructures can be employed to remove the organic dye pollutants in an inexpensive, straightforward manner. Lee et al. [127] synthesized Cu(I)-exchanged ZnS 1D nano-adsorbents as nanorods, nanobelts, and nanosheets, Figure 8a–c, via cation exchange of ZnS with CuCl (as the Cu(I) precursor). The surface charge and surface area of such nanostructures were tailored by changing the molar ratio of Cu to Zn. Accordingly, the molar ratio of 0.4 resulted in the highest negative surface charge, thus the most optimum cationic dye (Rh B) adsorption efficiency (86.6 mg·g^−1^) for the Cu-exchanged ZnS nano-adsorbents. The HRTEM images (Figure 8d,e) imply that the Cu exchanged ZnS nanorod is as crystalline as hexagonal ZnS with a comparable lattice spacing, yet with some stacking faults, arisen from replacement of smaller Zn^2+^ ions with larger Cu^+^ ones, which leads to lattice deformation. The successful exchange of the ions is further verified by the FFT pattern of the nanorod, Figure 8f, that shows the calculated lattice fringes comply well with the typical spacing values of the characteristic planes of the hexagonal ZnS. Figure 8g shows that depending on the adsorbent dosage and morphology, dye removal efficiency varies. The nanorods are able to offer the largest dye adsorption efficiency which remains fixed after 10 mg dosage. 

Mesoporous alumina nanofibers have also been synthesized through a combination of sol-gel, electrospinning, and calcination [128]. Possessing a significantly large surface area of 417.7 m^2^·g^−1^ and pore volume of 0.40 cm^3^·g^−1^ measured via the Brunauer–Emmett–Teller (BET) and the Barrett–Joyner–Halenda model, respectively, the nanofibers demonstrated a promising capability for adsorption of MO from water. To induce the mesoporosity, dendrimer polyamidoamine was a critical factor and supplied the hydrogen bonding necessary for the self-assembly of alumina-dendrimer-polymer.

Tian et al. [129] also synthesized NiCo_2_O_4_ nanorods for removal of MO from water. This composition is of interest for dye adsorption due to its abundance, low cost, and eco-friendliness. The nanocomposite nano-needles made of CdO/CdFe_2_O_4_ have shown favorable applicability for adsorption of CR dye by as much as 1491 mg·g^−1^ [130]. The porous nano-needles were synthesized simply by a sacrificial template of Cd_2_Fe(CN)_6_. In this system, the 1D morphology and porosity cooperatively offer an extensive surface area and a rich density of functional sites, and, thereby, render a large adsorption capacity.

#### 3.2.4. 1D Nanocomposite Nano-Adsorbents

Another 1D nanostructure is nanofibers that are typically made by electrospinning. The main distinction of this class of 1D nanomaterials compared to the previously mentioned ones is their length that could be even infinite, theoretically. The nanofibers that are suggested for dye adsorption can be classified to organic and inorganic ones. A more sophisticated class is nanocomposite nanofibers that could be as inorganic/organic, inorganic/inorganic, and even bio-nanohybrid involving a biopolymer as the secondary phase or as the host. With respect to the nanocomposite nanofiber-based adsorbents, Chen et al. [131] synthesized ZnO/SnO_2_ hybrid electrospun nanofibers (250 nm in diameter and multiple micrometers in length and mimicking a string of nanoparticles as small as 5–10 nm) for adsorption/photodecomposition of dye pollutants including MB, CR, MO, and ER. The electrospinning technique is a well-defined method for production of nanofibers in different arrangements (aligned, disordered, yarn etc.) and compositions (polymer, metal oxide, carbon, nanocomposite, biohybrid, etc.) [117,132,133,134,135,136,137]. Versus the traditional approaches such as chemical vapor deposition, hydrothermal, and template-assisted, electrospinning is an efficient, simple, and low-cost nanofiber production method. While ZnO nanofibers have proven to be efficient in the photodegradation of organics, mainly due to their large aspect ratio and extensive surface area, they suffer from quick recombination of electron hole pairs induced by light irradiation. Thus, the photogenerated electrons and holes must be adequately separated, e.g., by means of formation of hetero-structures. The ZnO/SnO_2_ nanofiber is one example for this kind of heterostructure that enables separation of electron-hole pairs, thereby, raising the photocatalytic efficiency for degradation of organic dyes. The fabrication procedure encompassing the sol-gel process and pyrolysis ensures formation of nanofibers with high surface porosity, which are promising for adsorption of water pollutants.

**Figure 9:**
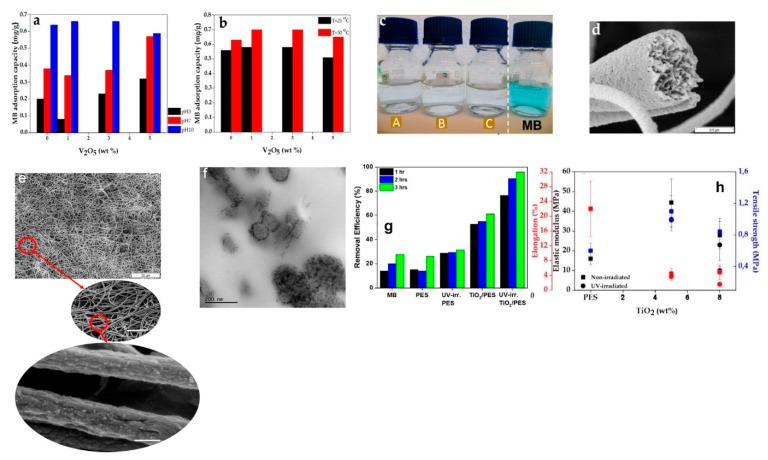
MB adsorption capacity for the V_2_O_5_/PES nanofibers with different nanofiller contents depending on (**a**) pH and (**b**) temperature (at pH 10). (**c**) The digital image shows the aqueous solutions decolorized by the V_2_O_5_/PES nanofiber adsorbents under a high temperature and pH compared to the untreated MB solution (A–C: exposed to PES, 1 wt.% V_2_O_5_/PES and 5 wt.% V_2_O_5_/PES nanofibers, respectively). (**d**) The SEM image witnesses the porosity available in the cross-section as well as on the surface of the V_2_O_5_/PES nanofibers (5 wt.%). Reproduced with permission [6]. Copyright 2016, MDPI. (**e**) The SEM image shows the morphology of the TiO_2_/PES nanofibers (8 wt.%) (from top to bottom the scale bars represent 5 and 0.2 µm, respectively). (**f**) The distribution mode of the TiO_2_ nanoparticles across the PES nanofibers’ cross section is shown in the TEM image. (**g**) The MB removal efficiency for the TiO_2_/PES (8 wt.%) nanofibers versus the neat ones induced by UV-irradiation (pH 10 and 9 mg·L^−1^ MB aqueous solution). (**h**) The uniaxial tensile test results witness the superior mechanical properties, i.e., elastic modulus and tensile strength, for the TiO_2_/PES nanofibers compared to the neat ones’. However, the nanocomposite nanofibers are less ductile and are brittle and upon UV-irradiation, they lose their enhanced properties down to the level of the neat nanofibers. Reproduced with permission [67].

Our group has also benefited from several inorganic/organic nanocomposite nanofiber adsorbents for MB removal [6,67]. For such studies, polyethersulfone (PES, a widely used polymer for construction of membranes for water treatment) has been chosen due to its remarkable thermochemical stability, and its outstanding robustness [25]. Additionally, PES’ isoelectric point ranges from 2.4 to 3.1, and, thus, under alkaline conditions, its surface is hydroxylated (negatively charged). This characteristic enables its large interaction with cationic dyes such as MB. Given the alkaline nature of the wastewater streams originated from industrial dyeing processes, thanks to the mentioned desirable features, PES, could be a good candidate for adsorption of the cationic dyes, particularly when formed as nanofibers. Noteworthy, PES films have been studied for the sake of adsorption of dye pollutants [138,139,140]. Yet, for the first time, we evaluated the applicability of the PES nanofibers with regards to dye adsorption. To apply the PES nanofibers even in the media with neutral and acidic pHs, a metal oxide nanofiller whose isoelectric point was inferior to that of PES was included into the nanofibers. Vanadium pentoxide (V_2_O_5_) is a metal oxide largely studied for optical switching devices, catalysis, solar cell, and sensors [141,142,143]. Its very low isoelectric point of 1–1.5 [144] renders a highly hydroxylated surface even at low pHs [145], and allows for an optimum interaction with cationic dyes. Accordingly, the PES nanofibers can be reinforced to operate in a wider pH spectrum by incorporating V_2_O_5_ nanoparticles. Figure 9a–c validate this postulate and show that the addition of the nanoparticles up to 5 wt.%, enables optimum removal of MB even under acidic and neutral conditions, which is comparable to the alkaline one. This high adsorption efficiency is partly contributed by the presence of small pores on the surface that facilitates capturing the dye molecules (Figure 9d). Despite the high potential of coupling photocatalytic nanoparticles with polymeric nanofibers offering a large reactive surface area and porosity for adsorption and photodecomposition of organic dye molecules, the likely photodegradation of the polymeric substrate should also be kept in mind. In another relevant study, we employed TiO_2_ nanoparticles to impart a photocatalytic effect to the PES nanofibers [67,146]. Figure 9e,f show the morphology of the TiO_2_/PES nanofibers, in particular, the distribution mode of the nanoparticles mainly accumulated on/near the ridge of the nanofibers. This style of residence of the nanoparticles is promising with respect to wettability and interaction with the dye molecules. As we showed, despite superhydrophilicity and efficient dye (MB) removal capacity (95%) of the nanocomposite nanofibers via adsorption/photodecomposition (Figure 9g), the thermomechanical properties are partly lost by UV irradiation (Figure 9h) [67]. Presumably, when O_2_ reacts with the electron/hole pairs originated from the CB and VB, respectively, reactive oxygen species such as O_2_^−^, ^1^O_2_, •O_2_H, and •OH form [147]. These reactive oxygen species can provoke a degradation process for the neighboring polymer chains that is likely extended to even deeper zones of the polymer structure. By penetrating and further reacting the carbon-centered radicals with the polymer chains, the chain cleavage is triggered [147].

Titanate nanotubes (TNTs) are one of the 1D nano-adsorbents that, due to their optimum stability, tubular structure, large surface area, ion exchange property, photoelectric function, and quantum size effect, have drawn attention for water treatment applications [148]. The TNT can partially adsorb dyes in a chemical manner and also via the electrostatic interaction between its surface negative charges with cationic dyes. Moreover, TNT’s photocatalytic ability can enable dye decomposition if it is improved via e.g., hybridization with GO. GO can raise the absorption of incident light and also photocatalytic effect by hampering the charge recombination. In this regard, GO acts as a photo-induced electron transmitter when hybridized with TNT. The TNTs@GO nano-adsorbent was able to remove 97.5% MB from water after 90 min of UV irradiation. According to the results of radical scavengers quenching tests and electron paramagnetic resonance (EPR) measurements, there are three main reactive elements including *h*^+^, •OH, and O_2_^·−^, that govern the photodegradation of MB. Additionally, the oxygen vacancy in TNTs@GO lowers the recombination rate of electron-hole pairs, and, thus, notably raises the photodegradation efficiency.

### 3.3. 2D Nano-Adsorbents

#### 3.3.1. 2D Carbonaceous Nano-Adsorbents

Graphene is a flat carbonaceous sheet derived from graphite, comprising a monolayer of carbon atoms arranged in a sp2 and sp3 hybridized honeycomb structure. Graphene oxide (GO), i.e., the oxidized form of graphene, is a high potential 2D adsorbent for dye removal. GO is synthesized through oxidation and then exfoliation of natural graphite flakes. For the former objective, graphite is subjected to strong oxidants, including KMnO_4_, KClO_3_, or NaNO_2_, alongside a strong acid, e.g., concentrated sulfuric acid or nitric acid. Afterward, exfoliation of the GO nanosheets takes place by ultrasonication. As a result, the GO nanosheets form that hold oxygen functional groups such as epoxides, alcohols, and carboxylic acids, and, thereby, react with functional water pollutants e.g., dyes, and disperse readily in water [149].

**Figure 10:**
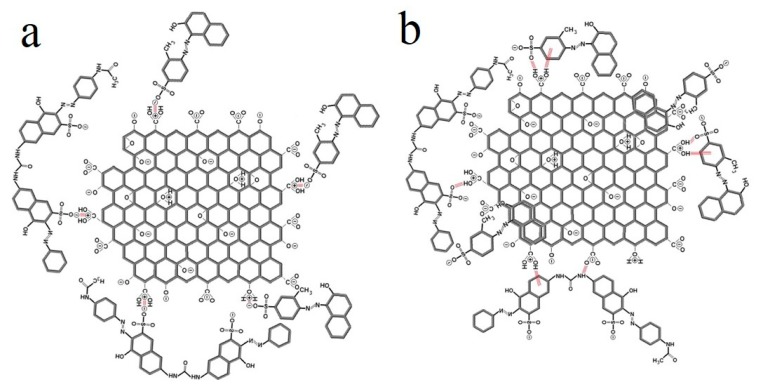
The AO8 and DR23 dyes are adsorbed onto GO via (**a**) electrostatic interaction between the protonated hydroxyl and carboxyl groups of GO and the anionic dyes, and via (**b**) H-bonding and π–π stacking. Reproduced with permission [150]. Copyright 2017, Elsevier.

Konicki et al. [150] used GO for adsorption of anionic azo dyes such as AO8 and DR23 from water. As the authors state, the electrostatic interaction between the anionic dyes and the positively charged functional groups, i.e., the protonated carboxyl and hydroxyl groups of GO is the underlying mechanism for adsorption of the azo dyes (Figure 10a). In addition to the electrostatic interaction, non-covalent bonding e.g., hydrogen bonding and π–π stacking, could also play a role due to the specific structure of GO. Figure 10b shows the hydrogen bond forming between the hydroxyl or carboxyl groups of GO and the oxygen atoms or aromatic ring of the dyes as the hydrogen electron donor and acceptor, respectively. Ultimately, the π electrons of the benzene group are potentially the proton acceptor and attract hydronium ions and form a hydrogen bond between the benzene group and hydronium ions. The π–π stacking can also take place between the bulk π systems on GO and the organics possessing benzene rings or C=C bonds. While the aromatic rings on the studied dye models encourage their adsorption via π–π stacking onto GO nanosheets. Their non-planar chemical structure largely declines this possibility. Overall, the authors attribute the large adsorption efficiency for the anionic dyes to the electrostatic interaction that is significant at an acidic pH due to the protonation of the GO’s oxygen containing functional groups. This finding has led to another opportunity for adsorption of cationic dyes that could be largely adsorbed to the GO nanosheets under alkaline conditions where the mentioned groups are deprotonated and highly negatively charged. They examined such a potential by using cationic dyes of BY28 and BR46 that are dissociated to the mehyl sulfate anion of CH_3_SO_4_^−^and the positively charged cationic dye whose positive charge is situated on a N atom.

Other than GO, reduced graphene oxide (rGO) nanosheets have also found applications with relevance to the adsorption of dyes from water. By applying chemical reduction, electro-reduction, thermal annealing, flash reduction, and enzymatic reduction, GO can be reduced as rGO that contains some remaining oxygen functional groups as well as structural defects [151]. Similar to GO but with a less density, the epoxy and hydroxyl groups are available on the basal plane, while the carboxyl and carbonyl groups on the edges. Specifically, rGO contains surface defects (vacancies) and sp^3^ bonded adatoms.

With respect to rGO, not only the defects and the available remaining functional groups facilitate the adsorption of dye molecules through electrostatic interaction and hydrogen bonding, but also the restored conjugated graphene structure enables interaction with aromatic structured organics. In general, rGO provides the possibility of π–π stacking, a hydrophobic interaction, or structural conjugation alongside the electrostatic interaction to adsorb a large range of dye models. On this subject, since 2015, there have been numerous studies implying the applicability of rGO for dye adsorption. For instance, Xiao et al. [152] developed a cysteine-modified rGO adsorbent that was able to adsorb anionic (i.e., IC) and cationic (i.e., NR) dyes mainly via π–π stacking. Possessing a plethora of oxygen functional groups, GO greatly adsorbs the cationic dye (NR) thanks to a strong electrostatic attraction. In contrast, it repels the anionic dye (IC) due to their same negative charge and lack of conjugated areas. Upon reduction by L-cysteine, the majority of the hydroxyl and epoxy groups present in the basal plane vanish while the carboxyl groups situated at the edge persist, which, thereby, imparts the Cys-rGO an optimum water dispersity as well as a conjugate structure. Such a specific feature enables adsorption of the anionic and cationic dye molecules on the nanosheets’ surface through the π–π stacking between the dyes’ delocalized π-bond and the adsorbent. Minitha et al. [153] studied the adsorption behavior of MB and MO on rGO experimentally and theoretically via the density functional theory (DFT). In this study, rGO shows a high but comparable adsorption efficiency for both cationic MB and anionic MO dyes, attributed to the present vacancies and/or conjugated structure of rGO, alongside electrostatic interaction.

Despite the previously mentioned merits of graphene derivatives for dye adsorption, their environmental implications should be also taken into account. In this regard, the fate, transformation, and toxicological effects of such nanomaterials in the environment have been widely introduced in some relevant precious articles [116,154,155]. With respect to the safety of graphene, dimensions, surface chemistry, and impurities play a decisive role and impact both mechanistic (aggregation, cellular processes, biodistribution, and degradation kinetics) and toxicological outcomes [116]. Therefore, for each class and specific composition of the graphene derivatives, systematic studies should be conducted to unravel the biological responses to graphene and their interplay with particular physicochemical properties (structure, surface, and colloidal properties). Such research results will be determining for the subsequent engineering and production of biocompatible graphene derivatives to be employed for water treatment that could indirectly affect human health.

#### 3.3.2. 2D Inorganic Nano-Adsorbents

In addition to the carbon-based 2D dye adsorbents, there are several other 2D nanostructures, mainly of an inorganic composition, that have also attracted attention for dye adsorption from water systems. In this regard, metal oxide nanomaterials such as NiO nano-disks have shown a promising potential for dye adsorption and photodecomposition. This metal oxide as doped and undoped has been synthesized via different techniques such as sol-gel [156], metal organic chemical vapor deposition [157], precipitation [158], solvothermal route [159], and a hydrothermal method [160]. The latter technique has been used for synthesis of NiO nano-disks (5–20 nm) as the photocatalysts that can photodegrade MB dye under visible light (98.7% in 20 min). Additionally, the existence of large surface microporosity for the nano-disks facilitates the degradation process of the dye molecules.

**Figure 11:**
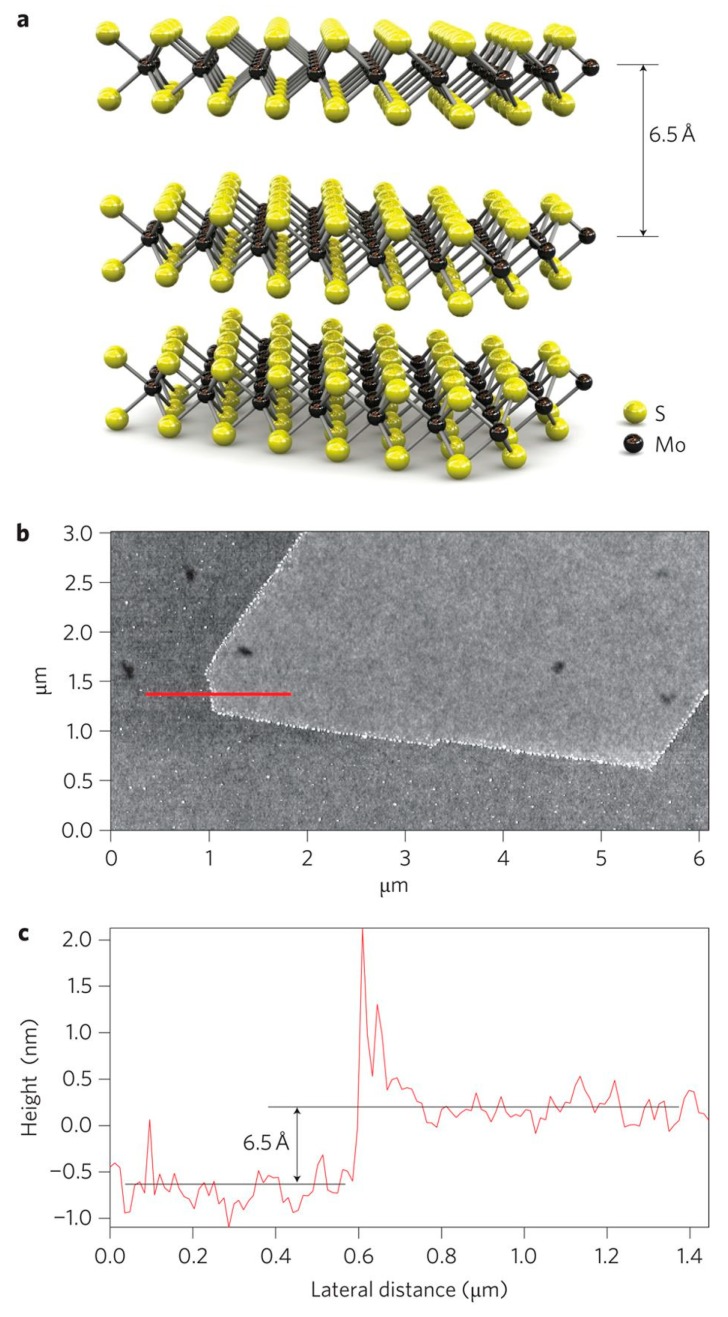
(**a**) 3D schematic illustration of the structure of MoS_2_, implying each single layer is 6.5 Å thick. Individual layers can be separated by scotch tape and via micromechanical cleavage. (**b**) AFM micrograph of a single MoS_2_ layer, overlaying a silicon substrate. (**c**) The cross-sectional plot monitored on the red line shown in b. Reproduced with permission [162]. Copyright 2011, Springer Nature.

Molybdenum disulfide (MoS_2_), which is a member of layered transition metal dichalcogenides (TMD), consists of a single Mo layer covalently sandwiched between two sulfur layers, i.e., creating an S–Mo–S layered structure. In contrast to the strong covalent bond existing in each MoS_2_ sheet, similar to graphite, there is a weak Van der Waals force between the adjacent layers (Figure 11a), which, thereby, facilitates exfoliation of the MoS_2_ few or monolayered nanoflakes from the bulk MoS_2_ [161]. The thickness of a monolayer MoS_2_ has been reported to be ~6.5 Å [162], Figure 11b,c, while a bilayer flake is 13 Å thick, i.e., two times of a monolayer MoS_2_’s [163]. Based on the experimental and theoretical studies, shrinking the dimensions of bulk MoS_2_ to a 2D monolayer transforms MoS_2_’s band gap to a direct one [164].

MoS_2_ nanosheets also show a remarkable adsorption efficiency for dye molecules particularly cationic dyes such as CB, MB, MV, and BB as well as RhB [165,166,167]. This high and mostly fast removal efficiency has been attributed to the release of [MoO_4_]^2−^ upon inclusion of MoS_2_ into the dye-containing aqueous solution. The [MoO_4_]^2−^ further reacts with cationic dye molecules, which engender prompt precipitation of a [(Dye^+^)_m_–([MoO_4_]^2−^)_n_] complex. Accordingly, the dye removal mechanism is, in fact, precipitation rather than adsorption [166]. With respect to the anionic dyes, on the other hand, the repulsion between the dye and [MoO_4_]^2−^ hinders precipitation and, thus, declines the removal efficiency [166].

The MoS_2_ nanosheets have also been applied as coupled with other nanomaterials. For instance, given their challenging recovery from aqueous solutions, via a hydrothermal process, they have been combined with magnetic nanoparticles such as Fe_3_O_4_ enabling their separation when exerting an external magnetic field. This nanohybrid adsorbent can show an adsorption capacity of 71 mg·g^−1^ for CR that reaches equilibrium in only 2 min [165]. MoS_2_ has been also coupled with rGO, which creates a 3D porous adsorbent with an extensive surface area of 44.4 m^2^·g^−1^ and a mean pore size of 35 nm. The main underlying mechanism for adsorption of CR on such a hierarchical adsorptive structure is assumed to be π–π stacking of the dye molecules on the nanocomposite [168]. MoS_2_/CuS nanosheet composite is another example of the hybrid 2D structures that benefits from a synergistic adsorption capacity, thereby, providing a promising adsorption efficiency for RhB (93.8%), MB (100%), and RhB 6G (84.73%), attributed to its high specific surface area (106.27 m^2^·g^−1^) and small mesopores (2.299 nm) [169]. With respect to the environmental applications of the MoS_2_ nanosheets including dye adsorption, the interested readers are referred to an excellent review [170].

Despite the mentioned merits of MoS_2_ in the removal of various dye pollutants, as stated earlier, dissolution of MoS_2_ in water and release of the molybdate ions can be considered a secondary pollution to the water streams and, thus, is not recommended.

Boron nitride (BN) nanosheets, which are also named as ‘white graphene,’ are another class of 2D nano-adsorbents that structurally comprise a number of hexagonal BN planes. They show exclusive properties such as a large band gap, electrical insulation, UV photoluminescence, remarkable thermal conductivity, resistance against oxidation, and chemical stability. Moreover, thanks to the BN link’s polarity that is enhanced at the extremely large surface area of *h*-BN nanomaterials, an optimum adsorption capability for dye molecules is expected. Induced by its large surface area, that is 1427 m^2^·g^−1^, and renders it as one of the materials with a very large surface area, porous BN nanosheets absorb ≈ 33 times their own weight due to their notable hydrophobicity, porosity, and swelling capacity. These materials are thermal, oxidation, and chemical resistant and can be recycled by burning, washing, and even heating in air [171]. It has been reported that the adsorption efficiency of the BN nanosheets is associated with their electronic properties and enhancement of this feature through nanohybridization with highly conductive metal nanoparticles such as Ag can bring about a superior adsorption capacity [172]. Accordingly, in a recent study [172], the BN nanosheets were surface decorated with Ag nanoparticles via a simple, one-pot pyrolysis method. Such nanostructures were employed as dye adsorbents for removal of RhB (880 mg·g^−1^). The existence of Ag nanoparticles on the BN nanosheets declines the BN’s external surface electron cloud density and, thereby, raises the electropositivity of B atoms and further enhances the interaction level with RhB. In fact, in this process, BN performs as the Lewis base because of the virtual orbitals of the B atom, while the dye acts as the Lewis acid due to the lone pair electrons in the O atoms.

As another 2D nano-adsorbent, the Bismuth-based layered nanostructures can be mentioned, which offer a suitable band gap for photocatalysis of the dye pollutants. Of the widely studied relevant compositions, Bi_2_O_3_, BiOX (X = F, Cl, Br, I), Bi_2_WO_6_, BiVO_4_, and Bi_2_MoO_6_ can be named [173,174,175,176,177,178,179,180]. BiOX is the most renowned for its safety, eco-friendliness, stability, and optimum photocatalytic effect. Such compounds, inherently, possess a unique layered structure wherein [Bi_2_O_2_]^2+^ blocks are separated by double slabs of halogen (X) atoms and create the [–X–Bi–O–O–Bi–X–] layered segments. This form of the structure governs an internal electric field that can optimally isolate the photogenerated electrons and holes emerging during the photocatalytic process [181]. Despite this advantage, having a large band gap (3.20–3.50 eV) [182], BiOCl can solely respond to UV light (taking only 5% of solar spectrum) and, as mentioned earlier, this limitation can narrow the application range for photocatalytic decomposition of organics. In contrast, BiOI and BiOBr are able to induce photocatalytic activity under visible light, but they suffer from recombination of photogenerated electrons and holes [183,184]. Accordingly, there have been a plethora of research studies directed to optimize the photocatalytic performance of BiOX materials such as hybridization as BiOCl/Bi_2_O_3_ [185], BiOBr/g-C_3_N_4_ [186], CdS/BiOI [187], Ag/AgX/BiOX [188], doping by inclusion of Mn, Fe, Ti, C etc. [182,189,190,191] and formation of solid solutions [192]. The latter has been the most attractive approach due to the capability of modulation of the band configuration and achievement of the best compromise between light absorption and redox potential [193]. This category of Bi-based compounds are mostly as 3D flower-like structures and will be presented in the next section. With respect to the 2D BiOX nanoadsorbents, Di et al. [191] doped BiOCl nanosheets with carbon quantum dots (CQDs) and scrutinized their photocatalytic efficiency in degradation of RhB under UV, visible, and near infrared (NIR) light irradiation. While BiOCl can photo-decompose only 48% RhB when exposed to UV irradiation, the CQD-BiOCl counterpart shows an enhanced photodegradation efficiency of up to 91% RhB under the same irradiation condition. Similarly, when subjected to NIR irradiation, the CQDs/BiOCl shows an enhanced removal efficiency versus BiOCl (40% Vs. 24% RhB, respectively). Regarding the visible light irradiation, a higher photodegradation efficiency was recorded for both the control and hybrid classes of BiOCl (containing 5 wt.% CQD), so that they offered 49% and 89% RhB removal efficiency, respectively. The CQDs raised the photocatalytic efficiency of the nanosheets mainly due to their excellent electron transfer ability, high light harvesting ability, and increased catalytic active sites.

### 3.4. 3D Nano-Adsorbents

**Figure 12:**
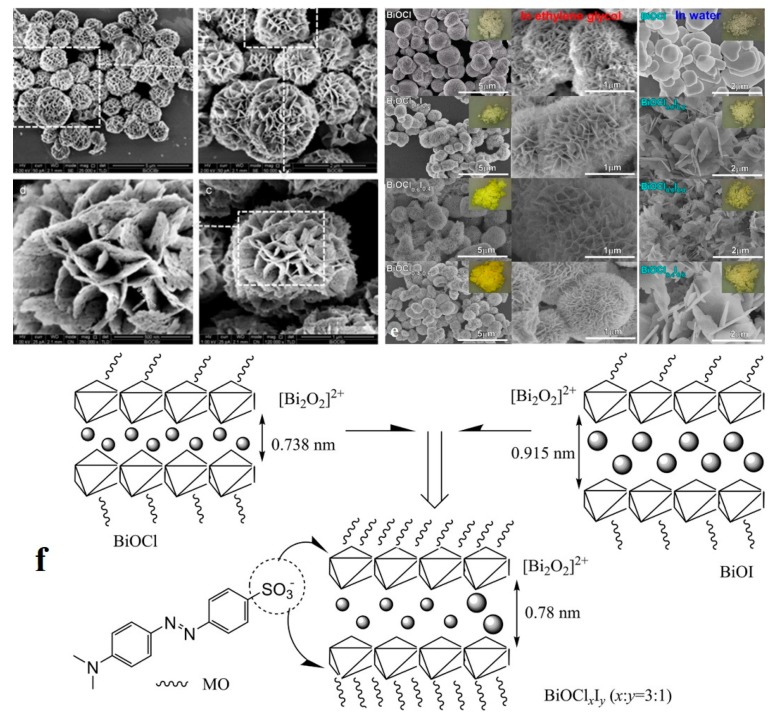
(**a**–**d**) FESEM images show the morphology of the BiOCl*_x_*Br_1−*x*_ flower-like 3D structures at different magnifications. Reproduced with permission [27]. Copyright 2015, John Wiley and Sons. (**e**) SEM images implying the morphology of BiOCl*_x_*I_1−*x*_ (*x* = 1, 0.8, 0.6, and 0.4) synthesized in ethylene glycol (the first two columns from left) and water (the last column). The inset camera pictures imply the color of the related samples. Reproduced with permission [197]. Copyright 2014, Elsevier. (**f**) Schematic illustration of the underlying mechanism for the enhanced MO adsorption in a BiOCl_x_I_y_ solid solution. Reproduced with permission [198]. Copyright 2016, Elsevier.

The hierarchical 3D nanostructures could also offer amazing facilities in terms of a functional surface area, which comprises a large number of adsorbing surfaces. They also raise the surface oxygen defects, thereby, lowering the chance of recombination of electron-hole pairs [180]. In this regard, 3D flower-like Bi-based solid solutions have shown excellent potential for photocatalytic degradation of organic water pollutants, particularly dyes. So far, a diverse range of bismuth oxyhalides solid solutions has been studied in relation to dye photo decomposition, e.g., BiOCl*_x_*Br_1-_*_x_* [27,183,194,195,196] (the 3D flower-like morphology is shown in Figure 12a–d), and BiOCl*_x_*I_1-_*_x_* [197,198,199] (the 2D plate-like and 3D flower-like morphology is shown in Figure 12e).

Yang et al. [180] synthesized 3D solid solutions made of BiOCl*_x_*Br_1-_*_x_* (*x* = 0–1) through a glycol- assisted hydrothermal treatment involving cationic polyacrylamide (C-PAM) and cetyltrimethylammonium bromide (CTAB). The as-developed materials were as hierarchical flower-shaped microspheres, which offer an extensive surface area and desirable routes for transfer and isolation of photogenerated electrons and holes. Additionally, tuning the ratio of Cl^−^ and Br^−^ engendered a reduction in the optical bandgap, which, thereby, enhanced the visible light absorption. Such solid solutions notably adsorbed and photodegraded MO when subjected to the visible light irradiation, particularly at *x* = 0.5. Xie et al. [199] also made hierarchical dahlia-like (solid solution) structures of BiOCl*_x_*I_1−*x*_ (*x* = 1.0, 0.75, 0.5, 0.25, 0) via a fast, inexpensive solid-state chemical approach. Similarly, the band gap could be adjusted by controlling the Cl/I ratio, thereby, optimizing the photocatalytic performance of the solid solutions. Among the various compositions studied, the dahlia-like BiOCl*_x_*I_1−*x*_ (x = 0.75) solid solution shows the best adsorption and photodegradation efficiency (98% in 1 h) for RhB when exposed to the visible light irradiation. In a related study, Zhang et al. [198] clarified the underlying mechanism for the bifunctional ability of the BiOCl_x_I_y_ solid solutions, i.e., adsorption followed by photodegradation of MO. As shown in Figure 12f, inclusion of a limited number of I atoms between the [Bi_2_O_2_]^2+^ layers of BiOCl enlarges the interlayer spacing (from 0.738 to 0.78 nm). As a result, the electron density in the resultant binary structure would decline. Such a circumstance is favorable for adsorption of MO, given that MO can be adsorbed via the van der Waals forces and by utilizing the electronegativity of SO_3_^−^ in neutral or alkaline conditions.

The 3D carbonaceous porous materials are another class of adsorbents that have shown desirable potential for dye removal from water. Constructed based on 2D building nano-blocks of GO and rGO, they can offer extraordinary adsorption capacity, fast adsorption kinetics, and the facility of engineering of the surface functionality coupled with the unique thermomechanical, and electrical properties [200,201,202,203]. In this regard, a variety of advanced chemistries has enabled scalable creation of 3D rGO macroporous structures in favorable shapes, sizes, and properties [204]. With its numerous active sites such as the oxygen-based functional groups, rGO is able to notably interact with the dye molecules, via hydrogen bonding as well as electrostatically. Moreover, the conjugated π electrons in sp^2^ carbon facilitate further interactions with the aromatic dye molecules [205,206]. There are several unique studies that challenge the dye adsorption efficiency of 3D rGO structures. For instance, Kim et al. [206] studied the adsorption behavior of AC1 and MB dyes on the 3D rGO macrostructures considering the isotherm and kinetic models. According to this research, adsorption of AC1 and MB complies better with the Freundlich and Langmuir isotherm models, respectively. However, they both follow the pseudo-second order kinetic model. With respect to the MB dye, the likely interactions are: (1) the electrostatic attraction between the amino groups and the oxygen-based functional groups that are positively and negatively charged, respectively, and (2) the π–π stacking that takes place between the localized π electrons of the conjugated aromatic rings for rGO and the MB molecules. In the case of AC1, the first interaction mode does not apply due to the presence of the negatively charged sulfonate (SO_3_^−^) groups that render them repulsive from the rGO surface. Yet, the AC1 molecules can be adsorbed on the rGO domains through π–π interaction and the hydrogen bonding. Particularly, the hydrogen bonding taking place between the AC1 molecules allows for adsorption and formation of stacked layers of them on the rGO surface, as verified by the Freundlich model. Graphene hydrogels (GHs), especially those doped with nitrogen and/or sulfur have also shown applicability for dye adsorption. In fact, the doping process has proved efficient in enhancement of catalytic, electrical, and chemical properties of the GHs [207,208]. Shi et al. [208] developed N/S GHs through an eco-friendly approach based on using glutathione as the binding and reducing material. The N/S-GHs can offer a promising adsorption capacity for various dye models including malachite green, MB, and CV. Structurally, the material encompasses distinct and interconnected 3D porous networks comprising many nanosheets that have been assembled as layers overlapping. Induced by the addition of glutathione, more intense π–π stacking takes place between the graphene nanosheets, while emergence of thiol and amine groups enlarges the spacing between the nanosheets and enables hydrogen and covalent bonding between the nanosheets. Additionally, the resulting structure turns out to be more hydrophobic. Other than glutathione, gelatin, i.e., an inexpensive, biodegradable water-soluble polypeptide, has also been proposed as a reducing and cross-linking agent for construction of the 3D graphene hydrogels. Gelatin possesses a plethora of amino and carboxyl groups that allow for cross-linking of the nanosheets as a 3D porous adsorptive structure [209]. The gelatin-reduced graphene oxide adsorbent shows excellent adsorption capacity for RhB, MB, and CV.

**Figure 13:**
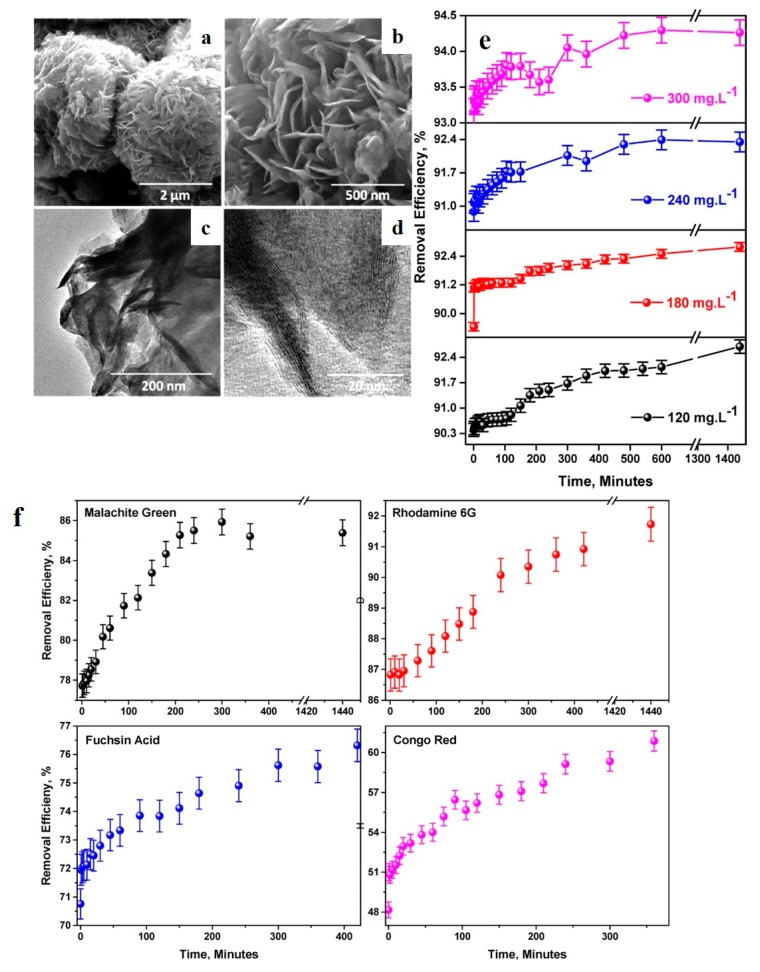
(**a**,**b**) FESEM and (**c**,**d**) HRTEM micrographs show the morphology and structure of the 3D MoS_2_ flowers. (**e**) The time-dependent MB removal percentage at various MB concentrations. (**f**) The time-dependent dye removal efficiency for the acidic and alkaline dye models. Reproduced with permission [210]. Copyright 2016, American Chemical Society.

In analogue to the graphene-based 3D structures, MoS_2_ nanosheets have also been assembled as 3D adsorbents for dye removal. In this regard, microspheres (as large as 1–5 µm) consisting of the MoS_2_ nanosheets have been synthesized based on a polyethylene glycol (PEG 200) template and challenged with respect to dye adsorption. Thanks to the hierarchical configuration of the MoS_2_ nanosheets (comprising 5–30 atomic thick lamellae of MoS_2_ held together by the Van der Waals forces), Figure 13a–d, the as-developed 3D MoS_2_ nano-adsorbent was able to efficiently remove (adsorb) 297, 204, 216, 183, and 146 mg g^–1^ MB, MG, rhodamine 6G, fuchsin acid, and CR dyes, respectively [210]. Figure 13e shows the MB dye adsorption efficiency versus time for the solutions with different MB concentrations. Notably, irrespective of the dye concentration, the adsorption took place fast and almost 90% of the dye was removed in only 10 min. The underlying mechanism for this adsorption behavior is assumed to be physiosorption induced by the weak Van der Waals forces or dipole-based interactions. Regarding other alkaline dye models including MG and Rh, there was a similar fast, efficient adsorption (Figure 13f). Among all the cationic dyes studied, removal efficiency for MB was the highest, which is followed by Rh. In contrast, the acidic dyes such as CR and FA showed a lower removal percentage by the nano-adsorbent, which implies that the electrostatic interaction also plays a crucial role in the adsorption process. More complicated hierarchical MoS_2_ nano-adsorbents have also been developed by several research groups. For instance, Ren et al. [211] loaded MoS_2_ flowers onto CoFe_2_O_4_ nanorods by electrospinning and then a hydrothermal treatment. This novel CoFe_2_O_4_/MoS_2_ heterostructure can photo-catalytically remove CR, MB, and MO when exposed to visible light. The cooperative action of the components, and the lower chance of recombination of photogenerated electron-hole pairs are the main causes for such an optimum dye removal efficiency. On the other hand, the magnetic properties of the nanorods enable recycling of the adsorbent at the end of the process. Li et al. [212] also synthesized a hierarchical structure consisting of the interlinked MoS_2_/CoAl-LDH (CoAl-layered double hydroxide) loaded carbon fibers via a bio-templating method. The precursor for the carbon fiber was the kapok fiber that was surface decorated with the MoS_2_ and CoAl-LDH nanosheets through a hydrothermal treatment. Such a unique porous structure could allow for adsorption and visible light driven photodegradation of CR. The flower-like GO/g-C_3_N_4_/MoS_2_ hybrids [213], N-TiO_2-x_@MoS_2_ [214], MoS_2_ nanosheet-coated TiO_2_ nanobelt [215], and Fe_3_O_4_-MoS_2_@Au [216] etc. are the other examples for the 3D hierarchical MoS_2_-based dye adsorbents.

Metal-Organic Frameworks (MOFs) are another class of 3D nano-adsorbents that allow for adsorption of organic dyes as well as their photodecomposition [217,218]. Such porous crystalline inorganic-organic hybrid materials offer desirable host–guest chemistry and also widely enable tuning of their porosity with respect to shape, functionality, and size. This flexibility in the porous structure is favorable for entrapment and adsorption of various pollutants, e.g., anionic or cationic dyes in a selective manner [219]. Additionally, MOFs’ crystalline structure facilitates comprehension of the underlying mechanism of adsorption inside the channels through X-ray crystallography. This fascinating ability of offering a visual insight into the applied framework, and also into the host–guest chemistry progressing within the channels of MOF, has enabled association of structure–functionality. Therefore, it has notably raised the knowledge level about MOFs [220].

Despite the above mentioned merits, MOFs are usually vulnerable to water, and this drawback has limited their applicability for water treatment purposes [221]. In recent years, a vast number of research studies has been devoted to address this bottleneck and improve water stability of MOFs. The adopted strategies can be based on (a) the hard/soft acid-base concept, employing divalent metal ions with nitrogen-containing ligands or high-valence metal ions with carboxylate-type ligands, (b) inclusion of hydrophobic groups in the ligand, (c) drastic reduction of the density of open metal sites (OMSs), and (d) application of biocompatible ligands [222]. The water-resistant MOFs have been frequently studied in the literature for the sake of dye adsorption. For instance, several MOFs were synthesized based on 1,4-benzenedicarboxylate (BDC) and 2-amino-1,4-benzenedicarboxylate (NH_2_-BDC) as organic linkers and tetraisopropyl orthotitanate as a metal source [223]. The as-developed MOFs were employed for ultrasound-assisted adsorption of multiple cationic dyes (Basic Red 46 (BR46), Basic Blue 41 (BB41), and MB). In another study [224], zeolitic imidazolate framework (ZIF-8) alone or as hybridized with GO and CNTs were used as adsorbents for removal of MG cationic dye. ZIF-8, ZIF-8@CNT, and ZIF-8@GO adsorbents could show maximum adsorption capacities of 1667, 2034, and 3300 mg g^−1^, respectively, at room temperature. Combining Mn^II^ ions, a multidentate carboxylate ligand, hydroxyl groups, and water, He et al. [225] synthesized an anionic microporous MOF. This MOF allowed a selective ion-exchange process of diethylammonium counter-cations by MB^+^ ions. From one hand, thanks to the negatively charged surface of the MOF, the neutral and anionic dyes such as Sudan I (SD^0^), solvent orange 7 (SO^0^), acid orange A (AO^−^) and MO^−^ are repelled. On the other hand, only the cationic dyes whose size is smaller than the MOF pore size are captured. This situation was not the case for Rhodamine 6G (R6G^+^) and Butyl Rhodamine B (BRB^+^) with a size exceeding the pore size of the MOF. Regarding adsorption-based removal of organic dyes by MOFs, an interested reader can refer to a number of relevant, useful reviews [217,226,227,228].

So far, a variety of the studied nano-adsorbents were introduced in terms of composition, dimensionality, adsorption mechanism, and production method. Table 2 summarizes and provides a list of such systems to assist the reader to acquire an informative overview of the state of the art in this field.

## 4. Conclusions and Future Perspectives

In this review, various classes of nanomaterials were introduced that can potentially offer advanced solutions for alleviation of the dye contamination problem in water streams, thereby, enabling water reuse. This issue is of paramount importance, bearing in mind the global water shortage crisis that is exacerbated daily. Particularly, those nanomaterials were considered that can show adsorptive properties and, at best, a combination of adsorptive and photocatalytic ones. Such nanomaterials were classified according to their dimensionalities (0–3D) and discussed in detail. Despite multiple merits of nanomaterials for dye removal including extraordinary specific surface area, faster removal kinetic, low sludge production, the possibility of use in diverse aqueous chemistries, cost efficiency, etc., the technology required for their employment in water treatment is still under development and at lab scale. Although there have been some pilot and full-scale field investigations based on the use of the nanoscale zerovalent iron (nZVI) for water treatment, other categories of water remediation techniques do not yet benefit from nanomaterials and this concept and idea has remained at a bench scale, proof-of-concept stage [37]. The conventional water treatment technologies are not necessarily more economical options in removal of various water pollutants, and particularly the ones existing at trace amounts. Additionally, many of these methods have reached their extreme of efficiency and could be unable to address the concerns related to the emerging pollutants and to meet the required water quality standards [229,230,231]. Additionally, the conventional techniques suffer from large energy consumption and production of a notable quantity of sludge and hazardous wastes. Conclusively, it is worth it to investigate the applicability of nano-adsorbents for dye decontamination due to the previously mentioned advantages in terms of energy and cost efficiency, particularly when reuse of nano-adsorbents is also taken into account. In this regard, there is a need to regeneration methods that enable reuse of the nano-adsorbents efficiently, safely, and economically, thereby, outperforming activated carbon. Despite such a necessity, recycling the adsorbents for further uses, to justify the related costs for production and integration have not been sufficiently scrutinized. Moreover, the nano-adsorbents have not been applied in realistic situations and for precise treatment of industrial wastewaters. Typically, the relevant research experiments done at lab scale consider only one type of dye and ignore co-existence of other dye, ionic, or organic pollutants, as seen in real dye wastewater, which compete for a limited number of available adsorption sites. Such a perspective was previously taken into account for activated carbon, and led to its commercialization. Technical difficulties with respect to scale-up and integration into a relevant technology, cost effectiveness, and energy-related issue are also hindering challenges that have delayed marketing of such products. For instance, TiO_2_ nanoparticles and CNTs are among the most widely studied nanomaterials for adsorption of dyes. However, they are toxic and produced in a costly manner involving high temperature and pressure. The former nano-adsorbent needs UV irradiation to photo decompose the dye pollutants that adds to the expenses of the treatment. In fact, it is highly necessary to produce large amounts of such nanomaterials at justifiable costs for water treatments, specific to different categories of wastewaters. Most importantly, environmental concerns regarding such small-scaled materials need to be properly addressed. The release of persistently lasting nanomaterials into various segments of the environment, e.g., water bodies, soil etc. where they can endanger the ecosystem and natural species must be controlled and hampered. In this regard, development of advanced systems that are able to precisely monitor the contamination level of water streams, e.g., in real time even by trace amounts of nanomaterials is of importance. Moreover, toxicity effect and mechanism of the nano-adsorbents in different shapes, sizes, and compositions need to be precisely investigated.

Nanomaterials are high potential candidate materials for water treatment and there is a promising prospect for their integration into point-of-use systems, and also in absolute removal of the current and emerging organic pollutants from water and wastewater.

## Abbreviation

a-CO*_x_*Oxygenated Amorphous CarbonAFMAtomic Force MicroscopyAOAcid OrangeAOPAdvanced Oxidation ProcessBBBasic BlueBETBrunauer–Emmett–TellerBNBoron NitrideBPBBromophenol BlueBRBasic RedC-PAMCationic PolyacrylamideCBConduction BandCBBCoomassie Brilliant BlueCNCCellulose NanocrystalCNTCarbon NanotubeCODChemical Oxygen DemandCQDCarbon Quantum DotCRCongo RedCSChitosanCSBChicago Sky BlueCTABCetyltrimethylammonium BromideCVCrystal VioletDDimensionDFTDensity Functional TheoryDRDisperse RedEREosin RedFAFuchsin AcidFESEMField Emission Scanning Electron MicroscopyFFTFast Fourier TransformGGraphiteGHGraphene HydrogelGOGraphene OxideHCPHyper-crosslinked-polymerHDAHexanediamineHRTEMHigh Resolution Transmission Electron MicroscopyICIndigo CarmineMBMethylene BlueMGMalachite GreenMOMethyl OrangeMOFMetal-Organic FrameworkMVMethyl VioletMWCNTMulti-walled Carbon NanotubenZVINanoscale Zerovalent IronNBBNapthol Blue BlackNIRNear InfraredNRNeutral RedPANPolyacrylonitrilePDAPolydopaminePEGPolyethylene glycolPEIPolyethyleniminePESPolyethersulfonePVDFPolyvinylidene fluorideRAPOPRich Amine Porous Organic PolymerRBBRemazol Brilliant BluerGOReduced Graphene OxideRhBRhodamine BROSReactive Oxygen SpeciesSEMScanning Electron MicroscopySET-LRPSingle-Electron Transfer Living Radical PolymerizationTEMPO(2,2,6,6-tetramethylpiperidin-1-yl)oxylTMDTransition Metal DichalcogenideTNTTitanate NanotubesTOCTotal Organic CarbonUVUltravioletVBValence BandZIFZeolitic Imidazolate Framework

## Figures and Tables

**Table 1:** Classification of dyes and their corresponding properties, applications, and toxicities (Reproduced with permission [5]. Copyright 2015, Elsevier).

Dyes	Properties	Applications	Toxicity	Examples
Acidic	Soluble in water, anionic	Nylon, wool, silk, paper, leather, ink-jet printing	Carcinogenic	Acid red 183, acid orange 10, acid orange 12, acid orange 8, acid red 73, acid red 18, sunset yellow, acid green 27, methyl orange, amido black 10B, indigo carmine
Cationic	Soluble in water, and liberates colored cations	Paper, PAN, treated nylons, treated polyesters, as antiseptic for biomedicine	Carcinogenic	MB, janus green, basic green 5, basic violet 10, rhodamine 6G
Disperse	Insoluble in water, non-ionic, for the aqueous/hydrophobic dispersions	Polyester, nylon, cellulose, cellulose acetate, acrylic fibers	Allergenic (skin), carcinogenic	Disperse orange 3, disperse red, disperse red 1, disperse yellow 1
Direct	Soluble in water, anionic, promotes wash fastness in case chelated with metal salts	Cotton, regenerated cellulose, paper, leather	Bladder cancer	CR, direct red 23, direct orange 39, direct blue 86
Reactive	Very high wash fastness thanks to its covalent bond with fiber, generates brighter colors compared to the direct dyes	Cotton, wool, nylon, ink-jet printing of textiles	Dermatitis, allergic conjunctivitis, rhinitis, occupational asthma	Reactive black 5, reactive green 19, reactive blue 4, reactive red 195, reactive red 198, reactive blue 19, reactive red 120
Vat	employs soluble leuco salts following reduction in an alkaline bath (NaOH)	Cellulosic fibers	-	Vat blue 4, vat green 11, vat orange 15, vat orange 28, vat yellow 20

**Table 2:** A summary of the diverse nanosized adsorbents introduced in this review.

Adsorbent System	Dimension-ality	Dye Models Studied	Adsorption Mechanism	Production Method	Reference
fungal chitosan nanoparticles	0D	RBB, MO, DR, NBB, CSB	electrostatic interaction	ionic gelation method	[42]
α-chitin nanoparticles	0D	MB, BPB, CBB	physical adsorption	chemical treatment of *Penaeus monodon* shell waste	[43]
cellulose nanoparticles in chitosan	0D	Rh	hydrogen binding and electrostatic interaction	freeze drying and compacting	[122]
Davankov-type hyper-crosslinked-polymer (HCP) nanoparticles	0D	MB, nigrosine, and AO	π–π stacking	emulsion polymerization then the Friedel–crafts crosslinking reaction using FeCl_3_ as the catalyst	[45]
Fe_2_O_3_, CoO, and NiO nanoparticles	0D	MB	ionic bonding	laser irradiation in the liquid for amorphization	[47]
Cr-doped ZnO nanoparticles	0D	MO	ionic bonding	solvothermal treatment	[48]
amino-coated Fe_3_O_4_ nanoparticles	0D	CR	π–π stacking, hydrogen binding, and electrostatic interaction	mussel-inspired polymerization	[56]
chitosan/Al_2_O_3_/magnetic iron oxide nanoparticle	0D	MO	electrostatic interaction	dispersion of iron oxide nanoparticles in aluminium isopropoxide/ethanol solution. The as-prepared core-shell nanoparticles were then dispersed in chitosan solution.	[10]
hollow cobalt nanoparticles	0D	MO	reductive degradation	A galvanic replacement reaction using aluminum nanoparticle templates	[102]
poly HEMA-CS-f-MWCNT	1D	MO	electrostatic interaction	functionalization of the nanotube with chitosan and polyHEMA	[112]
Fe_3_O_4_/CNTs	1D	sudan I, sudan II, sudan III, and sudan IV dye	electrostatic interaction	hydrothermal synthesis of Fe_3_O_4_ nanoparticles onto carbon nanotube	[113]
OMWCNT-κ-carrageenan-Fe_3_O_4_ nanocomposites	1D	MB	π–π stacking, hydrogen binding, and electrostatic interaction	chemical oxidation of CNTs and their functionalization with κ-carrageenan	[110]
a-CO*_x_*/G nanofilaments	1D	MB	electrostatic interaction and π–π stacking	electrospinning and carbonization of PAN nanofibers	[117]
Functionalized cellulose nanofibers	1D	CV	electrostatic interaction	functionalization of the cellulose nanofibers using Meldrum’s acid (2,2-dimethyl-1,3-dioxane-4,6-dione)	[26]
Cu(I)-exchanged ZnS 1D nanorods	1D	Rh B	electrostatic interaction	cation exchange of ZnS with CuCl	[127]
ZnO/SnO_2_ hybrid electro-spun nanofibers	1D	MB, CR, MO, and ER	photocatalysis	electrospinning, sol-gel process and pyrolysis	[131]
V_2_O_5_/PES nanofibers	1D	MB	electrostatic interaction	sol-gel and electrospinning	[6]
TiO_2_/PES nanofibers	1D	MB	Electrostatic interaction and photocatalysis	sol-gel and electrospinning	[67]
TNTs@GO	1D	MB	electrostatic interaction and photocatalysis	hydrothermal treatment	[148]
cysteine-modified rGO	2D	IC and NR	π–π stacking, and electrostatic interaction	hydrothermally (or hydrazine based) reduced GO	[152]
NiO nano-disks	2D	MB	photocatalysis	hydrothermal treatment	[160]
MoS_2_/rGO	2D	CR	π–π stacking	hydrothermal treatment	[168]
MoS_2_/CuS nanosheet	2D	RhB, MB, MO and RhB 6G	molecular diffusion, the van der Waals, and the electrostatic interactions	hydrothermal treatment	[169]
Ag/BN nanosheets	2D	RhB	lewis acid/base interactions	one-pot pyrolysis	[172]
BiOCl nanosheets doped with carbon quantum dots	2D	RhB	photocatalysis	solvothermal treatment	[191]
BiOCl*_x_*Br_1-_*_x_* (*x* = 0–1)	3D	MO	photocatalysis	glycol-assisted hydrothermal treatment	[180]
dahlia-like BiOCl_x_I_1−x_ (*x* = 0.75)	3D	RhB	photocatalysis	solid-state chemical approach	[199]
N/S-GHs	3D	MG, MB, and CV	π–π stacking, hydrogen, and covalent bonding	using glutathione as the binding and reducing material	[208]
3D MoS_2_	3D	MB, MG, rhodamine 6G, FA, and CR	physio-sorption induced by weak Van der Waals forces or dipole-based interactions, electrostatic interactions	synthesized based on a polyethylene glycol (PEG 200) template	[210]
MoS_2_ flowers onto CoFe_2_O_4_ nanorods	3D	CR, MB, and MO	photocatalysis	electrospinning and then hydrothermal treatment	[211]
